# Metabolite diversification via halogen salts enhances biocontrol potential of marine-derived *Trichoderma virens* B211

**DOI:** 10.1007/s13659-026-00634-y

**Published:** 2026-06-23

**Authors:** Minh Van Nguyen, Yeong Seok Kim, Seung Mok Ryu, Jaeyoung Kwon, Hun Kim, Gyung Ja Choi, Jae Woo Han

**Affiliations:** 1 Eco-Friendly New Materials Research Center, Korea Research Institute of Chemical Technology, Daejeon, 34114 Republic of Korea; 2 Herbal Medicine Resources Research Center, Korea Institute of Oriental Medicine, Naju, 58245 Republic of Korea; 3 KIST Gangneung Institute of Natural Products, Korea Institute of Science and Technology, Gangneung, 25451 Republic of Korea; 4 Department of Medicinal Chemistry and Pharmacology, University Science and Technology, Daejeon, 34113 Republic of Korea

**Keywords:** OSMAC strategy, Marine-derived fungus, *Trichoderma virens*, Secondary metabolites, Viridin derivatives, Gliotoxin derivatives, Plant disease control

## Abstract

In the search for natural products with potent activity against plant diseases, we employed the one strain–many compounds (OSMAC) strategy to the marine-derived fungus *Trichoderma virens* B211 by supplementing cultures with halogen salts such as sodium bromide (NaBr). Both solid and liquid cultures grown under NaBr-supplemented conditions exhibited markedly darker pigmentation of B211, a diversified secondary metabolite profile, and enhanced antibacterial activity against *Erwinia amylovora*. Bioassay-guided fractionation of these extracts led to the isolation of fourteen compounds (**1**–**14**), including two new polycyclic aromatic terpenoids (**1** and **5**) belonging to the viridin family. In vitro antimicrobial assays demonstrated that viridin (**3**) and *β*-viridin (**4**), together with gliotoxin (**8**), displayed broad-spectrum activity against diverse plant pathogenic fungi and bacteria. Notably, viridin derivatives **1** and** 2**, which are structural isomers, exhibited distinct bioactivity profiles: compound **1** exhibited antifungal activity, whereas compound **2** showed antibacterial activity. Among the isolated metabolites, only gliotoxin (**8**) and bisdethiobis(methylthio)gliotoxin (**9**) exhibited direct antibacterial activity against *E. amylovora.* Furthermore, NaBr-induced crude extracts in which compounds **8** and **9** were the major constituents, as well as the purified compounds themselves, effectively suppressed fire blight symptoms. Collectively, these findings highlight the effectiveness of the OSMAC approach in expanding the chemical diversity of metabolites produced by *T. virens* B211 and enhancing its potential as a biocontrol agent for plant disease management.

**Graphical Abstract:**

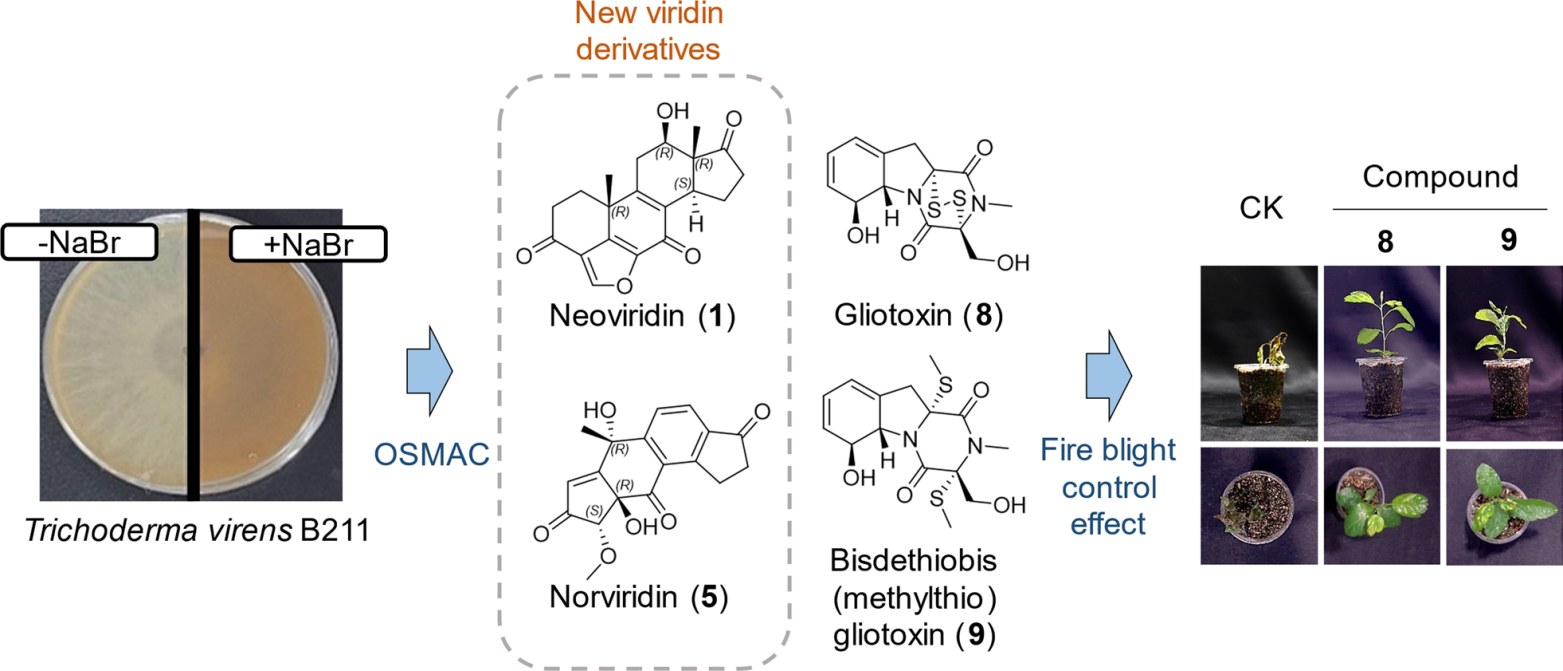

**Supplementary Information:**

The online version contains supplementary material available at 10.1007/s13659-026-00634-y.

## Introduction

Plant diseases caused by bacterial and fungal pathogens pose a significant threat to global agricultural productivity and food security [1]. Although synthetic fungicides and antibiotics have been widely used for crop protection, their prolonged and intensive application has raised serious concerns, including the emergence of resistant pathogens, environmental persistence, and negative impacts on non-target organisms [2]. These challenges have driven increasing interest in the discovery of natural products as sustainable alternatives for plant disease management [3, 4].

Marine-derived fungi have emerged as a prolific source of structurally diverse and biologically active secondary metabolites [5, 6]. Adaptation to unique marine environments, such as high salinity, pressure, and nutrient limitation, often leads to the evolution of distinct biosynthetic pathways and chemical scaffolds not commonly found in terrestrial microorganisms [6]. As a result, marine fungi have attracted considerable attention in bioprospecting programs aimed at identifying novel natural products with pharmaceutical and agrochemical potential [5].

Among both marine and terrestrial fungi, species of the genus *Trichoderma* are particularly well recognized for their roles as biological control agents in agriculture [7]. Their effectiveness in plant disease suppression is attributed to multiple mechanisms, including mycoparasitism, competition for nutrients and ecological niches, induction of plant systemic resistance, and the production of diverse antimicrobial secondary metabolites [8]. *Trichoderma virens* (Miller et al.) von Arx, in particular, is one of the most extensively studied species within the genus and is known to produce a variety of bioactive compounds, such as viridins, gliotoxin derivatives, and other polyketide- and peptide-based metabolites that contribute to its antagonistic activity against plant pathogens [9]. Several *T. virens*-based formulations have been developed for agricultural applications, underscoring its practical relevance in plant disease control [10]. Nevertheless, while terrestrial strains of *T. virens* have been intensively investigated, the chemical diversity and bioactive potential of marine-derived *T. virens* strains remain largely unexplored [11].

A major challenge in microbial natural product discovery is that many biosynthetic gene clusters remain silent or are weakly expressed under standard laboratory conditions, limiting access to the full metabolic capacity of a given strain [12]. The one strain–many compounds (OSMAC) strategy has emerged as an effective approach to overcome this limitation by systematically altering cultivation parameters to activate cryptic or low-abundance secondary metabolites [13]. Among various OSMAC-based manipulations, halogen salt supplementation has been shown to significantly influence fungal secondary metabolism, often resulting in enhanced chemical diversity and altered bioactivity profiles [14]. Such cultivation-based modulation provides a practical and scalable means of expanding the repertoire of bioactive metabolites without the need for genetic modification [15].

Bacterial plant diseases remain particularly difficult to control due to their rapid spread and the limited availability of effective bactericides [16]. In recent years, outbreaks of fire blight caused by *Erwinia amylovora* have become a major agricultural issue in South Korea, resulting in substantial economic losses and large-scale eradication of infected orchards [17]. Disease management has relied heavily on antibiotics and chemical bactericides, the use of which is increasingly constrained by regulatory restrictions and concerns over resistance development [18]. In this context, *E. amylovora* was selected as a key target organism in this study to evaluate the antibacterial potential of secondary metabolites produced by *T. virens* B211 in relation to plant disease control.

In the course of our ongoing screening program aimed at discovering bioactive natural products for plant disease management, we investigated the secondary metabolite production of the marine-derived fungus *T. virens* B211 using an OSMAC-based cultivation strategy. By supplementing culture media with halogen salts to modulate secondary metabolism, we observed pronounced changes in fungal pigmentation, metabolite diversity, and antibacterial activity against *E. amylovora*. Herein, we report the isolation and structural elucidation of fourteen secondary metabolites, including two new viridin analogues, and the evaluation of their antimicrobial activities against plant pathogenic bacteria and fungi. This study demonstrates the effectiveness of a halogen-based OSMAC approach in expanding the bioactive metabolite repertoire of marine-derived *T. virens* and highlights its potential as a source of natural products for plant disease control.

## Materials and methods

### Microorganisms and culture conditions

*T. virens* strain B211 was kindly provided by the Marine Fungal Resource Bank of Seoul National University, which was isolated from the mud of the tidal flats in Muan, Korea (35° 01′ 39.5″ N 126° 25′ 17.1″ E). *E. amylovora* TS3128 was provided by Rural Development Administration (Wanju, Korea). Potato dextrose agar/broth (PDA/PDB; BD Difco) and tryptic soy agar/broth (TSA/TSB; BD Difco) were used to maintain and culture fungi and bacteria, respectively. All plant pathogenic fungi and bacteria used in the minimum inhibitory concentration (MIC) test were listed in Table S1. Bacterial species were maintained on TSA at 30 °C, except for *Pseudomonas syringae* pv. *actinidiae* and *Xanthomonas arboricola* pv. *pruni*, which were grown at 25 °C and 28 °C, respectively. Fungal species were maintained on PDA at 25 °C, except for *Botrytis cinerea, Cylindrocarpon destructans*, and *Phytophthora infestans*, which were grown at 20 °C.

### OSMAC cultivation

To isolate the active compounds under OSMAC conditions, B211 was cultured in liquid and solid media. Mycelial disks (5 mm in diameter) obtained from the edge of B211 colonies were prepared as inoculum using a sterile cork borer. For liquid culture, ten mycelial disks were inoculated into a 2 L baffled Erlenmeyer flask containing 400 mL of PDB and incubated at 25 °C with shaking at 160 rpm for 14 days. For solid culture, ten mycelial disks were evenly spaced around the PDA plate (150 mm in diameter; SPL Life Sciences, Pocheon, Korea) and incubated at 25 °C for 14 days. To verify the effectiveness of the OSMAC strategy, halogen salts (NaBr, NaCl, or KI) were added to the medium at 3% (w/v) as needed.

### Extraction and isolation of compounds from liquid and solid cultures

The liquid culture was harvested by centrifugation at 10,000×*g* for 20 min, filtered through Whatman No. 1 paper, and subsequently extracted twice with equal volumes of ethyl acetate. The solid culture was cut into 1 × 1 cm pieces, transferred to a 5 L glass beaker, and extracted by soaking in methanol overnight. After centrifugation and filtration, the methanol extract was concentrated *in vacuo* to remove methanol, and then extracted twice with equal volumes of ethyl acetate. Anhydrous Na_2_SO_4_ was added to remove the residual water in ethyl acetate extracts. Two types of ethyl acetate extracts from liquid and solid cultures were filtered, dried, and designated as LE and AE, respectively.

The entire process of isolating compounds **1–14** from LE and AE is shown in a flow chart in Fig. S1. Each extract was prepared from PDB (8 L) and PDA (6 kg) cultures supplemented with 3% NaBr (w/v). LE (1.1 g) and AE (2.0 g) were subjected to medium pressure liquid chromatography using a Biotage Isolera One system (Uppsala, Sweden) equipped with a SNAP KP-Sil cartridge (25 g; Biotage) via gradient elution with dichloromethane-methanol (100:0 to 50:50 v/v). Thin-layer chromatography (TLC) analysis was performed using a Kieselgel 60 F_245_ and RP–18 F_245S_ plates (0.25 mm thickness; Merck Millipore, Burlington, MA, USA). According to the TLC pattern, the elutes from LE and AE were each divided into six fractions as follows: LE1–LE6 and AE1–AE6.

The active fraction LE3 (356.0 mg) was applied to a Sephadex LH-20 column (25–100 μm; Cytiva, Marlborough, MA, USA) and eluted with methanol. The active fraction was subsequently subjected to preparative high-performance liquid chromatography (HPLC) using a Shimadzu LC-20AR system (Kyoto, Japan) equipped with a Capcell Pak C18 column (250 × 20 mm, 5 μm; Shiseido, Tokyo, Japan). A stepwise gradient elution from water to acetonitrile at a flow rate of 5 mL/min afforded pure compounds **1** (7.5 mg), **2** (13.1 mg), **3** (7.2 mg), **4** (13.9 mg), **5** (6.8 mg), **6** (9.4 mg), **7** (23.7 mg), and **8** (27.2 mg).

Fractions of AE3 (580.8 mg) and AE4 (384.4 mg) were purified by methanol elution on a Sephadex LH-20 column, and then subjected to preparative HPLC using a linear gradient of 30–50% acetonitrile in water at a flow rate of 5 mL/min. This procedure afforded pure compounds **8** (2.0 mg), **9** (190 mg), **10** (3.0 mg), **11** (3.0 mg), **12** (19.7 mg), **13** (5.5 mg), and **14** (3.4 mg). Compound **8**, identical to that obtained from LE3, was also isolated from AE4.

### Spectroscopic analyses

Nuclear magnetic resonance (NMR) spectra were recorded on Bruker Avance spectrometers operating at 400 and 500 MHz spectrometers (Bruker, Billerica, MA, USA). The isolated compounds (**1**–**14**) were dissolved in deuterated solvents including methanol-*d*_4_ (99.8 atom % D), chloroform-*d* (99.8 atom % D), and DMSO-*d*_*6*_ (99.9 atom % D) (Cambridge Isotope Laboratories, Tewksbury, MA, USA). Chemical shifts were referenced to the residual solvent peaks: *δ*_H_ 3.31 ppm and *δ*_C_ 49.00 ppm for methanol-*d*_4_; *δ*_H_ 7.26 ppm and *δ*_C_ 77.16 ppm for chloroform-d; and *δ*_H_ 2.50 ppm and *δ*_C_ 39.52 ppm for DMSO-*d*_*6*_. High-resolution electrospray ionization mass spectrometry (HRESIMS) was recorded using a Synapt G2 mass spectrometer (Waters, Milford, MA, USA). Electronic circular dichroism (ECD) spectra were recorded on a JASCO J-715 spectropolarimeter (Tokyo, Japan).

### Computational analysis

The absolute configuration of compound **5** was determined through a comprehensive computational study, following established protocols [19, 20]. Conformational searches were initially performed using Spartan′14 software (Wavefunction, Irvine, CA, USA) with the molecular mechanics force field. Conformers within a cumulative Boltzmann distribution cutoff of 0.95 were selected and further optimized using Gaussian 09 software (Wallingford, CT, USA) at the B3LYP/6-31G + (d,p) level. ECD spectra were calculated using time-dependent density functional theory at the CAM-B3LYP/SVP level with a polarizable continuum model (PCM, solvent: methanol). Theoretical spectra were simulated with a half-bandwidth of 0.3 eV, and the resulting curves were processed using SpecDis 1.71 software for comparison with experimental data. After geometry optimization, gauge-including atomic orbital shielding constants were calculated at the B3LYP/6–311 + G(d,p) level in the PCM (DMSO) solvent model. To further validate the stereochemical assignment, DP4 + probability analysis was applied to compare calculated and experimental NMR chemical shifts, providing strong statistical support for the proposed configuration.

### Broth microdilution assay for in vitro antimicrobial activity

The MIC values of extracts or compounds against various plant pathogenic bacteria and fungi were determined using a two-fold dilution assay in 96-well plates. Briefly, fungal spores or bacterial cells were added to the wells to give a final concentration of 1 × 10^4^ cells/mL. To perform a two-fold dilution assay with an initial concentration of 200 μg/mL or more, the crude extracts and purified compounds were dissolved in dimethyl sulfoxide (DMSO) at the concentration of 40 mg/mL. Antibiotics (streptomycin sulfate, oxytetracycline, and cycloheximide) and 1% DMSO were used as positive and negative controls, respectively. After 1–3 days of incubation, the MIC value was determined by visual observation. Bacterial cell growth of *E. amylovora* was assessed by measuring OD_600_ with a xMark microplate spectrophotometer (Bio-Rad, Hercules, CA, USA), and inhibition rate (%) was calculated as (1 − OD_600_ of treatment/OD_600_ of control) × 100.

### Disease control efficacy of these extracts and active metabolites

The fire blight control efficacy of extracts (LE and AE, obtained from PDB and PDA cultures supplemented with 3% NaBr) and compounds (**8** and **9**) was evaluated using Chinese pearleaf crab apple (*Malus asiatica* Nakai) seedlings. Briefly, 4–5-week-old seedlings grown in a greenhouse at 25 ± 5 °C were sprayed with extracts (500 μg/mL in 1% DMSO) and compounds (20, 100, or 500 μg/mL in 1% DMSO). Streptomycin (100 μg/mL) and 1% DMSO were used as positive and negative controls, respectively. After drying overnight at room temperature, the plants were spray-inoculated with *E. amylovora* (4 × 10^8^ CFU/mL). The inoculated plants were placed in a dew chamber for 2 days, and then transferred to a growth chamber (25 ℃, 80% relative humidity, 12-h photoperiod). After 7 days, the disease severity index was assessed using the modified method of Wang et al. (2010). The disease severity index ranged from 0 to 10 as follows: 0, no symptoms; 1, partial necrosis of the shoot tip; 2, complete necrosis of the shoot tip; 5, complete necrosis on the petiole of terminal leaves; 10, complete necrosis on the main stem.

### Statistical analysis

For the quantitative data of this study, analysis was performed at least two times independently with three biological replicates unless indicated. Statistical significance was evaluated using one-way ANOVA followed by the Student *t*-test or Tukey's honestly significant difference (HSD) (*P* < 0.05).

## Results

### NaBr supplementation enhances antibacterial activity and metabolite diversity in *T. virens*

**Fig. 1:**
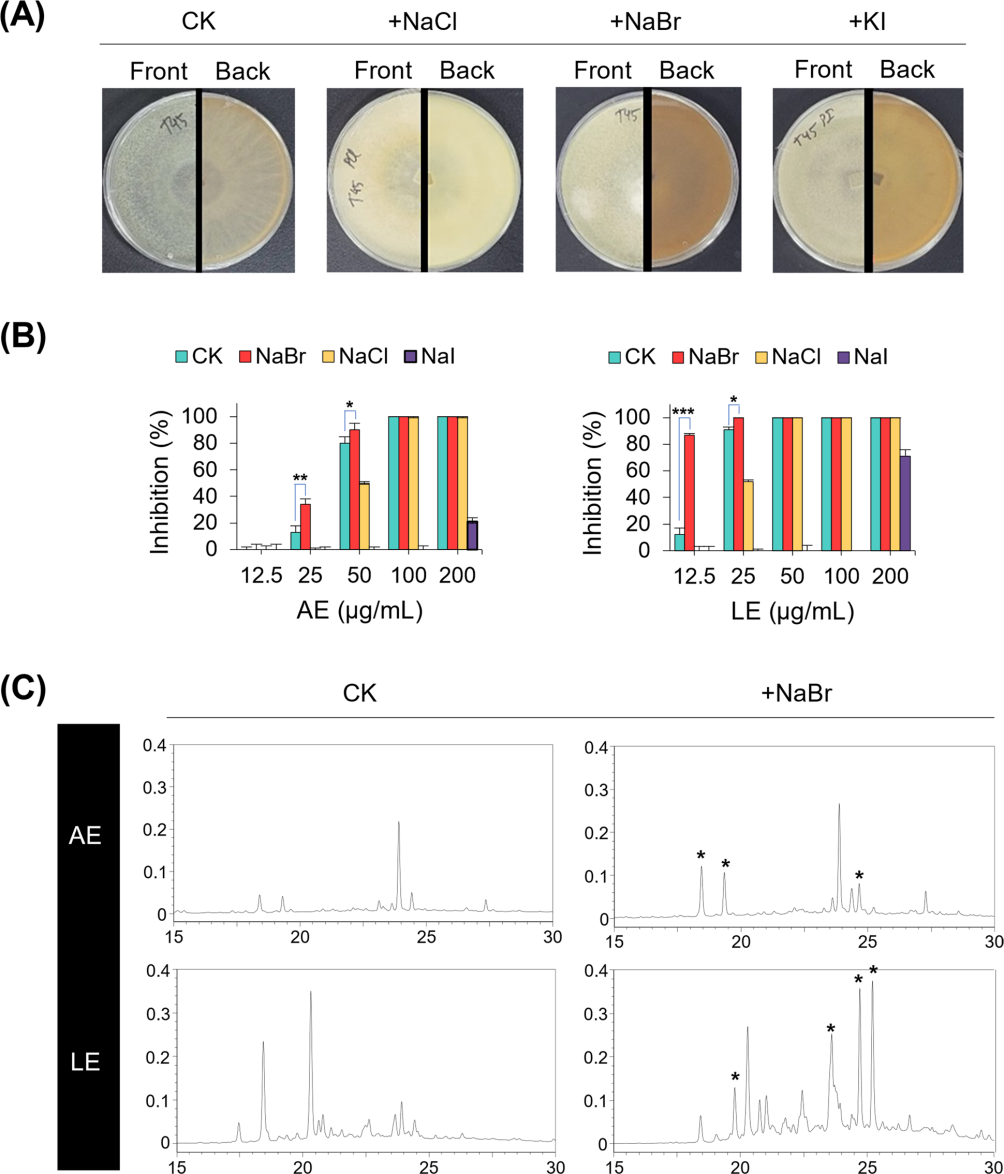
Effects of halogen salt supplementation on *Trichoderma virens* B211. **A** Colony morphology on potato dextrose agar supplemented with 3% halogen salts (NaCl, NaBr, and KI) versus control (CK). **B** Antibacterial activity of ethyl acetate extracts from agar (AE) and liquid (LE) cultures against *Erwinia amylovora*, with or without 3% halogen salts. Data are shown as mean ± SD. Different letters above the bars indicate statistically significant differences (Student *t*-test; *P < 0.05; **, *P* < 0.01; and ***, *P* < 0.001). **C** HPLC chromatograms of secondary metabolites under different culture conditions, showing that NaBr supplementation markedly increased metabolite diversity, especially in liquid culture

Since the isolated strain originated from a marine environment, it is likely to produce diverse secondary metabolites upon culture under halogen salt stress. The growth of the marine-derived *T. virens* strain B211 was investigated in response to the addition of three different halogen salts on solid (agar) culture conditions. Supplementation with 3% halogen salts altered the pigmentation of B211 colonies (Fig. 1A). When NaBr was added, B211 colonies secreted a darker brown pigment (Fig. 1A). Fungal growth was not significantly affected by the addition of NaCl and NaBr; however, KI supplementation resulted in a slower growth rate without complete inhibition (data not shown).

Ethyl acetate extracts obtained from agar and liquid cultures (AEs and LEs) were evaluated for their antibacterial activity against *E. amylovora.* As shown in Fig. 1B, when cultured with the addition of NaBr, the activity of AE and LE was significantly increased. In contrast, addition of NaCl decreased the activity of AE and LE compared to CK (without halogen salt). The addition of KI severely impaired antibacterial activity (Fig. 1B). Overall, LEs completely inhibited the growth of *E. amylovora* at lower concentrations (MICs of 25–50 μg/mL) than AEs (MICs of 100 μg/mL). In particular, the LE from NaBr-containing PDB medium showed the most potent activity against *E. amylovora* (MIC = 12.5 μg/mL). HPLC analysis revealed differences in the production of secondary metabolites between the solid and liquid cultures (Fig. 1C). When cultured with 3% NaBr supplementation, B211 produced a variety of secondary metabolites, which was particularly maximized under liquid culture conditions (Fig. 1C). When the effects of NaBr concentration on activity were investigated, LEs of PDB supplemented with 1%, 3%, and 6% NaBr showed MIC values of 15.6, 12.5, and 32 µg/mL, respectively (Table S2). HPLC analysis further revealed that increasing NaBr supplementation in the liquid medium led to greater diversification of metabolites in the LEs (Fig. S2).

The enhanced antibacterial activity observed in NaBr-supplemented cultures correlated well with pronounced changes in metabolite profiles, particularly under liquid culture conditions. Liquid extracts consistently exhibited stronger activity against *E. amylovora* than agar extracts, indicating that submerged fermentation favors the biosynthesis or accumulation of bioactive metabolites in B211. Notably, while increasing NaBr concentrations promoted greater chemical diversification, maximal antibacterial activity was achieved at an intermediate supplementation level (3%), suggesting that optimal bioactivity depends not only on metabolite diversity but also on the relative abundance and composition of active constituents.

### Isolation and identification of secondary metabolites from *T. virens*

**Fig. 2:**
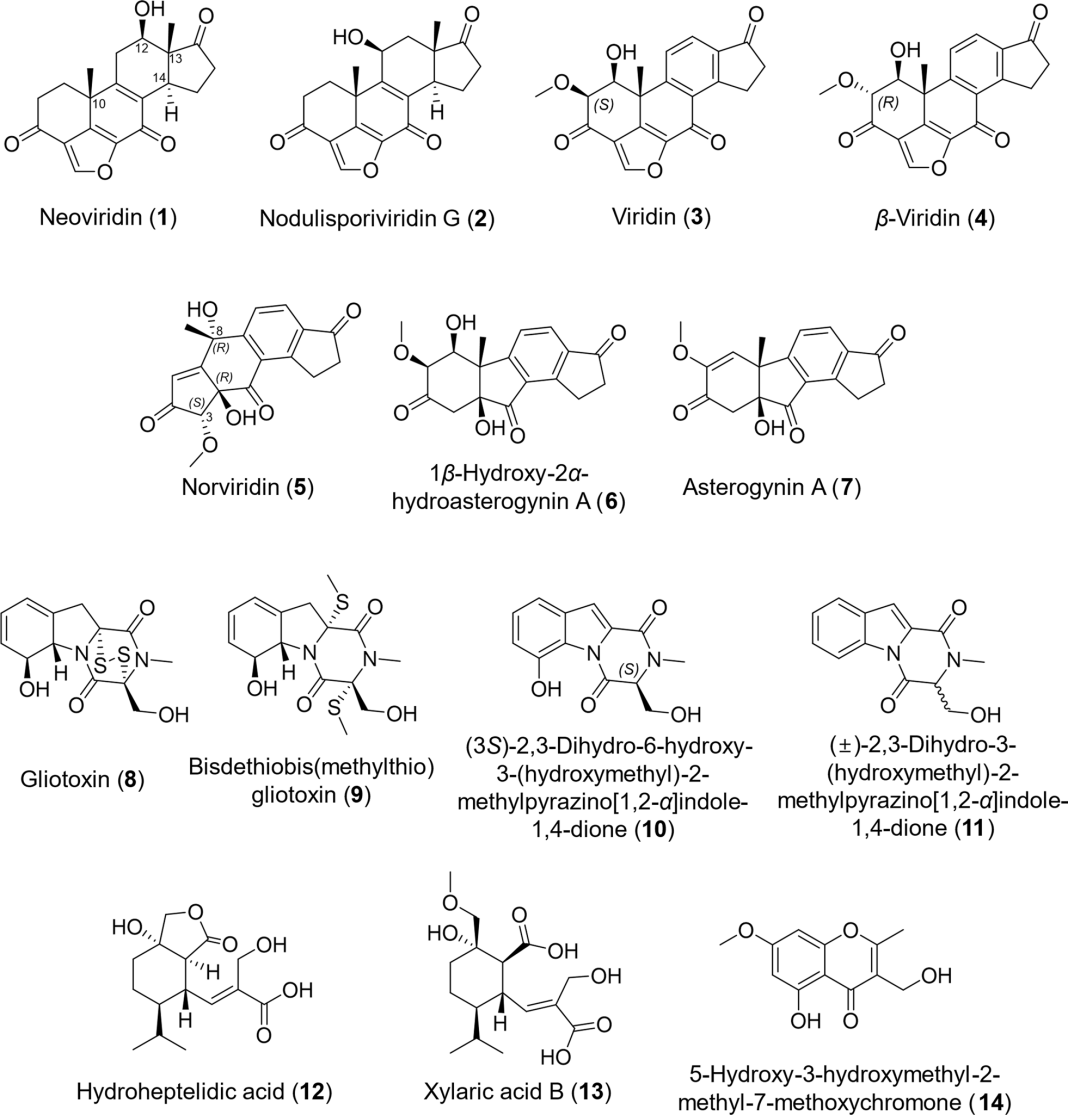
Chemical structures of compounds **1**–**14** isolated and identified from *Trichoderma virens* B211 under culture conditions supplemented with 3% NaBr. Compounds **1**–**8** were obtained from liquid culture, whereas compounds **8**–**14** were derived from agar culture. Notably, compound **8** was isolated from both liquid and agar cultures

From large-scale cultures grown on PDA (6 kg) and PDB (8 L) supplemented with 3% NaBr, fourteen compounds (**1–14**) were purified (Figs. S1 and S3). Spectroscopic analyses and comparison with literature data identified two new metabolites (**1** and **5**) and twelve known ones, including nodulisporiviridin G (**2**), viridin (**3**), *β*-viridin (**4**), 1*β*-hydroxy-2*α*-hydroasterogynin A (**6**), asterogynin A (**7**), gliotoxin (**8**), bisdethiobis(methylthio)gliotoxin (**9**), (3*S*)−2,3-dihydro-6-hydroxy-3-(hydroxymethyl)−2-methylpyrazino[1,2-*α*]indole-1,4-dione (**10**), (±)−2,3-dihydro-3-(hydroxymethyl)−2-methylpyrazino[1,2-*α*]indole-1,4-dione (**11**), hydroheptelidic acid (**12**), xylaric acid B (**13**), and 5-hydroxy-3-hydroxymethyl-2-methyl-7-methoxychromone (**14**) [21–31]. The chemical structures of **1–14** are shown in Fig. 2.

Compound **1** was obtained as white wax. The HRESIMS analysis (Fig. S4) revealed quasi-molecular ions at *m/z* 341.1391[M + H]^+^ and 363.1207 [M + Na]^+^ (calculated m/z 341.1389 and 363.1208 for C_20_H_21_O_5_ and C_20_H_20_O_5_Na, respectively), indicating the molecular formula of C_20_H_20_O_5_ with eleven degrees of unsaturation. The ^1^H and ^13^C NMR spectra, along with COSY and HSQC spectra of **1** (Figs. S5–S8), revealed the presence of two methyls, five aliphatic methylenes, an aliphatic methine, an olefinic methine, an oxygenated methine, and ten quaternary carbons, including two aliphatic quaternary carbons at *δ*_C_ 37.9 and 51.0 ppm; five *sp*^2^ at *δ*_C_ 122.1, 132.9, 145.5, 146.7, and 160.9 ppm; and three ketone carbons at *δ*_C_ 174.0, 191.5, and 221.3 ppm (Table 1).

**Table 1:** ^1^H and ^13^C NMR data of compounds **1** and **5** measured at 400 MHz

No	Compound 1 in CDCl_3_		Compound 5 in DMSO-*d*_*6*_	
	*δ*_H_, mult. (*J* in Hz)	*δ*_C_, mult	*δ*_H_, mult. (*J* in Hz)	*δ*_C_, mult
1	1.96, td (13.2, 5.1) 2.27, overlapped	32.9, CH_2_	6.36, s	128.2, CH
2	2.29, overlapped 2.85, overlapped	36.7, CH_2_	–	202.7, C
3	–	191.5, C	4.21, s	80.9, CH
4	–	122.1, C	–	79.7, C
5	–	146.7, C	–	195.4, C
6	–	145.5, C	–	137.8, C
7	–	174.0, C	–	155.6, C
8	–	132.9, C	–	71.4, C
9	–	160.9, C	–	179.2, C
10	–	37.9, C	7.94, d (8.4)	127.1, CH
11	2.43, ddd (19.7, 9.0, 3.1) 2.88, overlapped	33.3, CH_2_	8.00, d (8.4)	129.0, CH
12	4.20, dd (9.0, 7.4)	69.6, CH	–	127.2, C
13	–	51.0, C	–	157.5, C
14	2.69, overlapped	43.3, CH	3.50, m	27.9, CH_2_
15	2.16, m 3.16, ddd (13.3, 7.4, 5.3)	23.0, CH_2_	2.70, ddd (6.8, 4.8, 1.6)	36.6, CH_2_
16	2.62, dd (19.6, 7.4) 2.71, ddd (19.6, 5.3, 2.1)	36.6, CH_2_	–	205.9, C
17	–	221.3, C	1.79, s	33.4, CH_3_
18	0.97, s	7.7, CH_3_	3.63, s	59.8, CH_3_
19	1.61, s	27.3, CH_3_		
20	8.12, s	147.5, CH		

**Fig. 3:**
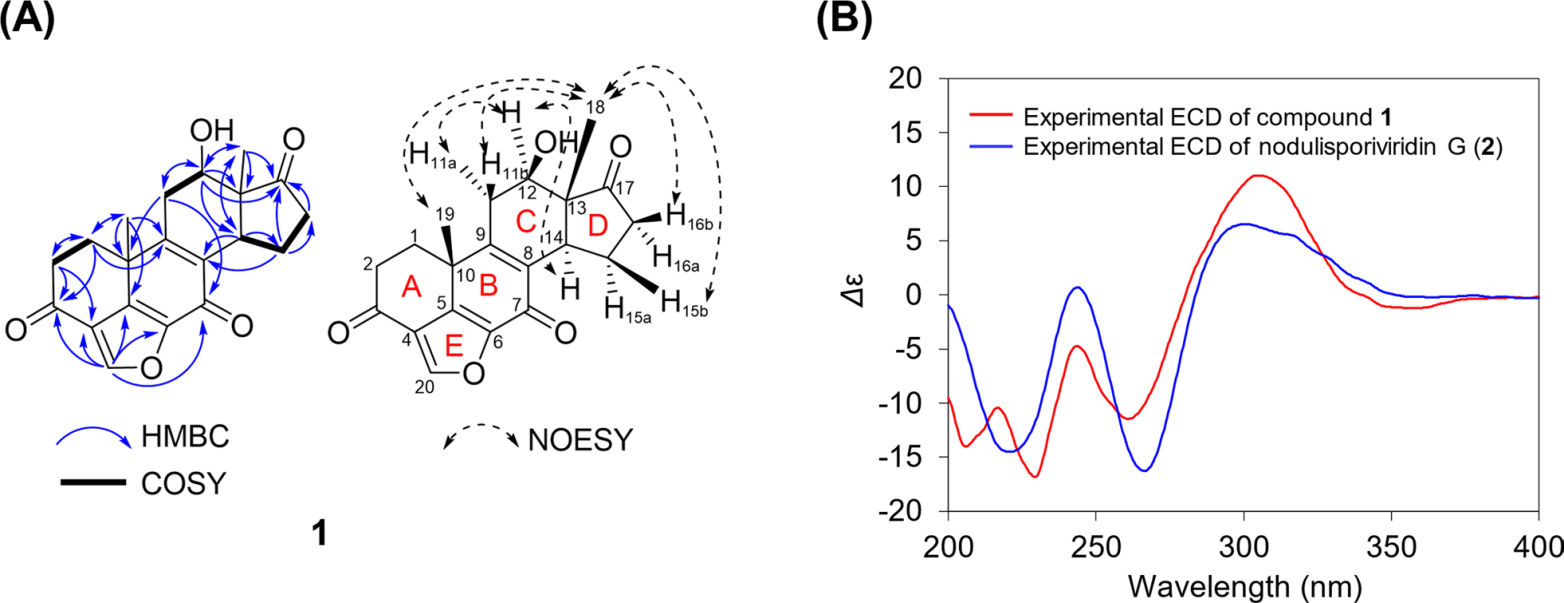
Structure determination of compound **1** by NMR and ECD analyses. **A** Key COSY (bold lines), HMBC (blue solid arrows), and NOESY (black dashed arrows) NMR correlations. **B** ECD spectra of compound **1** and nodulisporiviridin G (**2**)

The structure of compound **1** was established using ^1^H–^13^C HMBC and ^1^H–^1^H COSY spectra (Figs. 3A, S7, and S9). COSY correlations of H-1/H-2, H-11/H-12, and H-14/H-15/H-16 defined partial fragments. The structures of rings A and E were confirmed by HMBC correlations from H-1 to C-2, C-3, and C-19; from H-2 to C-1, C-3, and C-4; from H-19 to C-1, C-5, and C-10; and from H-20 to C-3, C-4, C-5, and C-6 (Fig. 3A). The structures of the rings C and D were supported by correlations from H-11 to C-12; from H-12 to C-11, C-13, C-14, C-17, and C-18; from H-14 to C-8, C-15, and C-18; from H-15 to C-8, C-16, and C-17; from H-16 to C-17; and from H-18 to C-12, C-13, C-14, and C-17 (Fig. 3A). Correlations from H-1 and H-19 to C-9 and from H-11 to C-10 indicated that rings A and C are connected through the C-9/C-10 bond (Fig. 3A). Weak correlations from H-11 and H-20 to C-7 suggested that rings E and C ring are linked through the ketone carbon C-7, forming the B ring and completing the planar structure of compound **1** in agreement with the degree of unsaturation (Fig. 3A).

The relative stereochemistry of **1** was determined from NOESY experiments. Because of overlapping signals in the ^1^H NMR spectrum, 1D-NOESY was employed to observe correlations between irradiated and enhanced signals. Irradiation of the C-18 methyl protons enhanced the H-11β, H-15β, H-16β, and H-19, while irradiation of the oxygenated proton H-12 enhanced the H-11α and H-14 (Figs. 3A and S10). These data indicated that H-11α, H-12, and H-14 are oriented in the same direction, whereas protons H-11β, H-15β, H-16β, H-18, and H-19 are oriented oppositely, similar to the pattern observed in compound **2** (Fig. S10). Thus, the relative stereochemistry of compound **1** was assigned as 10R*,12R*,13R*,14S* (or the enantiomeric configuration) (Fig. 3A).

Given the similarity of the planar structure and relative configuration of compound **1** to nodulisporiviridins G (**2**) and H, the absolute configuration was further determined by ECD spectroscopy (Fig. 3B). The ECD spectrum of compound **1** exhibited negative Cotton effects (CEs) at 220 and 260 nm and positive CE at 310 nm, closely matching those of nodulisporiviridins G (**2**) and H [21]. Accordingly, the absolute configuration of the new compound **1** was established as 10*R*,12*R*,13*R*,14*S*, and it was named neoviridin.

Compound **5** was obtained as yellow wax. The HRESIMS analysis (Fig. S11) showed a quasi-molecular ion at *m/z* 329.1020 [M + H]^+^ and 351.0841 [M + Na]^+^ (calculated *m/z* 329.1026 and 351.0845 for C_18_H_17_O_6_ and C_18_H_16_O_6_Na, respectively), indicating the molecular formula of C_18_H_16_O_6_ with eleven degrees of unsaturation. The ^1^H and ^13^C NMR spectra, along with COSY and HSQC spectra of compound **5** (Figs. S12–S15), revealed the presence of a methyl, a methoxy, two aliphatic methylenes, three olefinic methines, an oxygenated methine, and ten quaternary carbons, including two oxygenated quaternary carbons at *δ*_C_ 79.7 and 71.4 ppm; five *sp*^2^ at *δ*_C_ 127.2, 137.8, 155.6, 157.5, and 179.2 ppm; and three ketone carbons at *δ*_C_ 195.4, 202.7, and 205.9 ppm (Table 1).

**Fig. 4:**
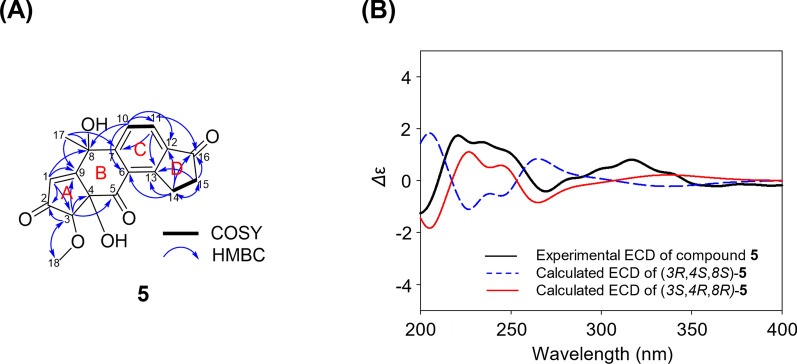
Structure determination of compound **5** by NMR and ECD analyses. **A** Key COSY (bold lines) and HMBC (blue solid arrows) NMR correlations. **B** Experimental vs. calculated ECD spectra of *3R*,*4S*,*8S*- and *3S*,*4R*,*8R*- stereoisomers, confirming stereochemical assignment

The ^1^H–^1^H COSY correlations of H-10/H-11 and H-14/H-15 (Fig. 4A) defined partial fragments of compound **5**. The connectivity of ring A was confirmed by HMBC correlations from H-1 to C-2, C-3, and C-9 and from H-3 to C-2, C-4, and C-9 (Fig. 4A). A methoxy group was assigned to C-3 based on the correlations from H-3 to C-18 and from H-18 to C-3. The structures of rings C and D were supported by HMBC correlations from H-10 to C-11, C-12, and C-6; from H-11 to C-7, C-13, and C-16; from H-14 to C-6, C-12, C-13, C-15, and C-16; and from H-15 to C-13, C-14, and C-16 (Fig. 4A). In addition, strong HMBC correlations were observed from H-1 to C-8; and H-10 to C-8; from H-3 to C-5; and from H-17 to C-7, C-8, and C-9, together with weaker correlations from H-10 to C-5 (Fig. S16). Integration of these data with the degree of unsaturation indicated that C-4 and C-6 are connected through the ketone carbon C-5, while C-7 and C-9 are linked via a quaternary carbon C-8, which carries a methyl group (C-17) and a hydroxy group. These features completed the B ring and established the planar structure of compound **5**, as shown in Fig. 4A.

The absolute configuration of compound **5** was determined through a combined analysis of ECD and DP4 + NMR chemical shift calculations. Considering the presence of three stereogenic centers, eight possible stereoisomers were generated: (*3R*,*4R*,*8R*)-, (*3S*,*4S*,*8S*)-, (*3R*,*4R*,*8S*)-, (*3S*,*4S*,*8R*)-, (*3R*,*4S*,*8R*)-, (*3S*,*4R*,*8S*)-, (*3S*,*4R*,*8R*)- and (*3S*,*4R*,*8S*)-**5**. were generated. Comparison of the calculated and experimental ECD spectra revealed that the (*3S*,*4R*,*8R*)-**5** stereoisomer exhibited the closest resemblance to the observed curve, followed by (*3S*,*4R*,*8S*)-**5** (Figs. 4B and S17). To further distinguish between these two possibilities, ^1^H and ^13^C NMR chemical shift calculations were performed. The calculated chemical shifts of (*3S*,*4R*,*8R*)-**5** showed superior agreement with the experimental data compared to those of (*3S*,*4R*,*8S*)-**5**, as confirmed by DP4 + probability analysis (Table S4). Based on these combined computational and spectroscopic results, the absolute configuration of compound **5** was unambiguously established as *3S*,*4R*,*8R*. The newly identified compound was subsequently named norviridin.

Compound **11** was obtained as white wax. The low-resolution ESIMS spectrum (Fig. S18) displayed a quasi-molecular ion at *m/z* 244.9 [M + H]^+^. The ^1^H NMR spectrum in DMSO-*d*_*6*_ (Fig. S19) showed five olefinic protons at *δ*_H_ 8.35 (d, *J* = 8.3 Hz, 1H), 7.78 (d, *J* = 7.7 Hz, 1H), 7.52 (ddd, *J* = 8.3, 7.2, 1.3 Hz, 1H), 7.41 (td, *J* = 7.7, 1.3 Hz, 1H), and 7.35 (s, 1H) ppm; three oxygenated protons at *δ*_H_ 4.53 (t, *J* = 2.3 Hz, 1H), 4.00 (dd, *J* = 11.6, 2.8 Hz, 1H), and 3.87 (dd, *J* = 11.6, 2.0 Hz, 1H) ppm; and a *N*-methyl protons at *δ*_H_ 3.04 (s, 3H) ppm. The ^13^C NMR and DEPT spectra of compound **11** (Fig. S19) revealed thirteen carbons, including one *N*-methyl carbon, one oxygenated methylene carbon, one aliphatic methine carbon, five olefinic methine carbons, and five quaternary carbons. The chemical shift of the olefinic protons and carbons supported the presence of a 1,2-disubstituted benzene ring. Two carbonyl carbons at *δ*_C_ 165.2 and 156.3 ppm indicated a diketopiperazine moiety. Comparison of the ^13^C NMR data of compound **11** with literature values (Table S5) led to its identification as (±)−2,3-dihydro-3-(hydroxymethyl)−2-methylpyrazino[1,2-*α*]indole-1,4-dione [29]. The specific rotation [*α*]_D_^20^ (*c* 0.1, methanol) was measured to be zero, indicating that compound **11** is a racemic mixture. To the best of our knowledge, this is the first report of compound **11** being isolated from a natural source.

### Antimicrobial activity of compounds 1–14 against plant pathogens

The isolated compounds **1–14** were evaluated for their activity to inhibit the growth of plant pathogenic bacteria and fungi using the microdilution assay (Tables 2 and 3). Among the furanosteroids (**1–4**), viridin and *β*-viridin (**3** and **4**) exhibited broad-spectrum antibacterial activity with MICs of 0.4–50 μg/mL and antifungal activity with MICs of 25–100 μg/mL. Neoviridin (**1**) exhibited antifungal activity against *A. brassicicola*, *C. coccodes*, *P. infestans* (MICs = 25–50 μg/mL), whereas its structural isomer, nodulisporiviridin G (**2**), displayed antibacterial activity against *A. citrulli* and *R. solanacearum* (MICs = 50 μg/mL). Interestingly, most of steroid-like compounds **1–7**, except for norviridin (**5**), were effective in inhibiting the growth of *P. infestans* (MICs = 0.4**–**200 μg/mL). Gliotoxin (**8**), an epipolythiodioxopiperazine featuring a 2,5-diketopiperazine ring system bridged by a transannular disulfide bond, displayed the most potent activity against all bacterial and fungal pathogens, with MIC values ranging from 0.8 to 25 μg/mL. In contrast, bisdethiobis(methylthio)gliotoxin (**9**), lacking the disulfide bridge, showed markedly reduced activity, being active only against *E. amylovora* and *C. coccodes* (MICs = 200 μg/mL). Other diketopiperazine derivatives (**10** and **11**) exhibited weak or no antifungal activity, with MICs of 200**–**400 μg/mL. Compounds **12–13** showed no activity against all tested plant pathogens at concentrations up to 400 μg/mL.

**Table 2:** Minimum inhibitory concentrations (MICs) of the isolated compounds **1–14** against plant pathogenic bacteria

Bacterial pathogen	MICs (μg/mL) of compounds^a^						
	**1**	**2**	**3**	**4**	**8**	**9**	**PC**^**b**^
*Acidovorax citrulli*	–^c^	50	25	50	12.5	–	0.2
*Agrobacterium tumefaciens*	–	–	100	100	25	–	0.4
*Burkholderia glumae*	–	–	50	100	12.5	–	1.6
*Dickeya chrysanthemi*	–	–	100	100	12.5	–	0.4
*Erwinia amylovora*	–	–	–	–	3.2	200	0.4
*Pectobacterium carotovorum* subsp. *carotovorum*	–	–	50	100	12.5	–	0.4
*Pseudomonas syringae* pv.* actinidiae*	–	200	25	50	3.2	–	0.2
*Pseudomonas syringae* pv. *lachrymans*	–	–	–	–	25	–	3.2
*Ralstonia solanacearum*	200	50	25	25	12.5	–	0.8
*Xanthomonas campestris* pv.* campestris*	–	200	50	50	25	–	3.2
*Xanthomonas arboricola* pv.* pruni*	–	200	50	50	6.3	–	1.6

^a^Active compounds were neoviridin (**1**), nodulisporiviridin G (**2**), viridin (**3**), *β*-viridin (**4**), gliotoxin (**8**), and bisdethiobis(methylthio)gliotoxin (**9**); inactive compounds (**5–7** and **10–14**) were not shown. ^b^Oxytetracycline was used as a positive control (except for *E. amylovora*, where streptomycin sulfate was used as a positive control). ^c^MIC > 400 μg/mL

**Table 3:** Minimum inhibitory concentrations (MICs) of the isolated compounds **1–14** against plant pathogenic fungi

Fungal pathogen	MICs (μg/mL) of compounds^a^									
	**1**	**2**	**3**	**4**	**6**	**7**	**8**	**9**	**11**	**PC**^**b**^
*Alternaria brassicicola*	50	–^c^	12.5	0.4	–	–	6.4	–	–	6.4
*Alternaria porri*	–	–	25	25	–	–	25	–	–	12.5
*Botrytis cinerea*	–	–	25	25	–	–	12.5	–	–	25
*Cladosporum cucumerinum*	–	–	12.5	12.5	–	–	25	–	–	12.5
*Colletotrichum coccodes*	50	–	0.4	0.4	–	–	1.6	200	–	3.2
*Cylindrocarpon destructans*	–	–	12.5	1.6	–	–	3.1	–	400	12.5
*Fusarium oxysporum*	400	–	6.4	1.6	–	–	12.5	–	–	25
*Fusarium fujikuroi*	–	–	12.5	6.4	–	–	12.5	–	–	12.5
*Fusarium graminearum*	–	–	3.2	0.8	–	–	1.6	–	200	6.4
*Magnaporthe oryzae*	–	–	100	50	–	–	12.5	–	400	3.1
*Phytophthora infestans*	25	12.5	0.4	1.6	200	50	0.8	–	–	0.8
*Stemphylium vesicarium*	–	–	6.4	6.4	–	–	1.6	–	–	3.1

^a^Active compounds were neoviridin (**1**), nodulisporiviridin G (**2**), viridin (**3**), *β*-viridin (**4**), 1*β* -hydroxyl-2*α*-hydroasterogynin A (**6**), asterogynin A (**7**), gliotoxin (**8**), bisdethiobis(methylthio)gliotoxin (**9**), and (±)−2,3-dihydro-3-(hydroxymethyl)−2-methylpyrazino[1,2-*α*]indole-1,4-dione (**11**); inactive compounds (**5**, **10**, and **12–14**) were not shown. ^b^Cycloheximide was used as a positive control. ^c^MIC > 400 μg/mL

### Disease control efficacy of extracts and gliotoxin derivatives against fire blight

**Fig. 5:**
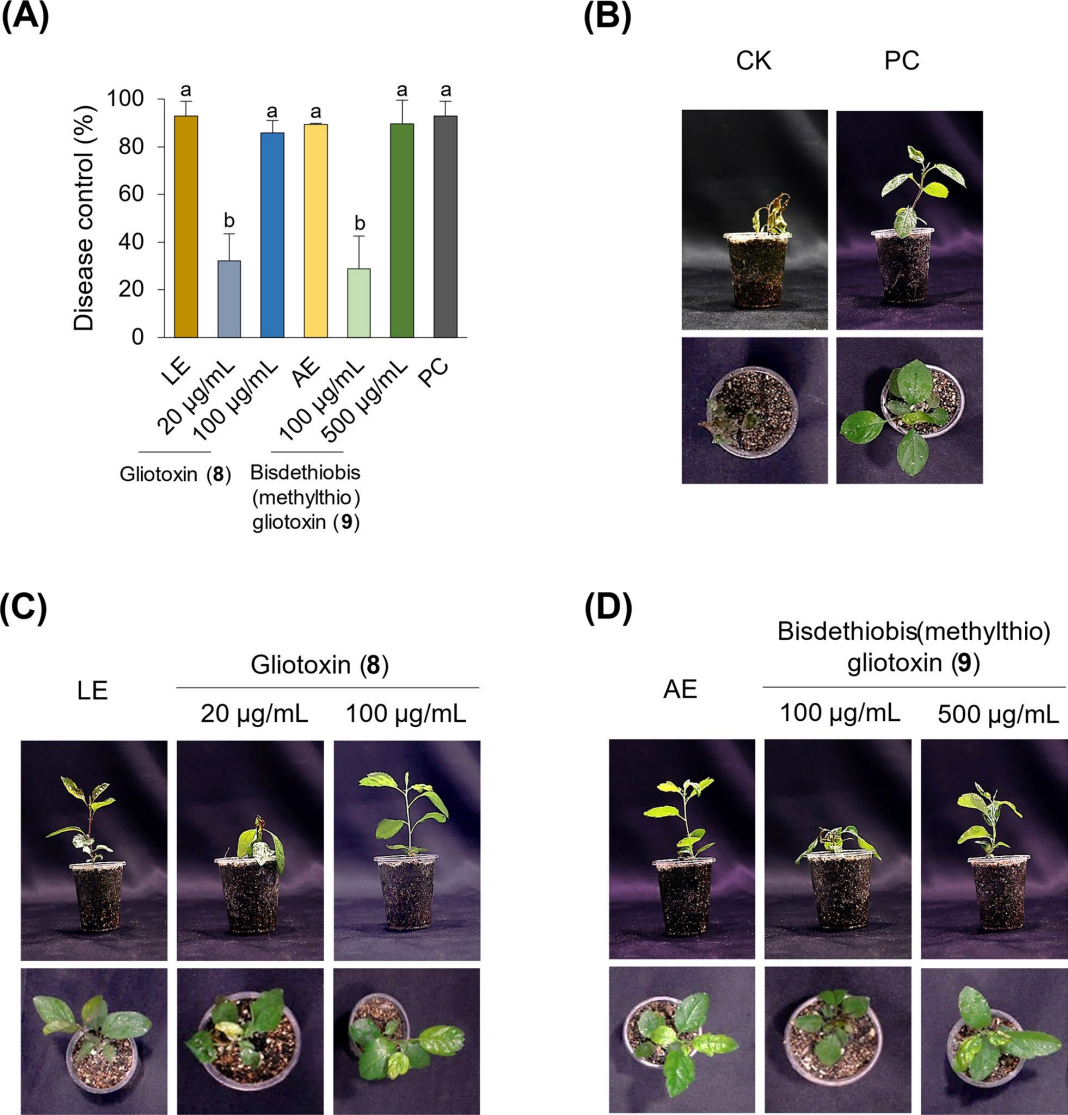
**A** Fire blight control efficacy of crude extracts (LE and AE, 500 μg/mL) and their major active compounds, gliotoxin (**8**) (20 and 100 μg/mL) and bisdethiobis(methylthio)gliotoxin (**9**) (100 and 500 μg/mL), against *E. amylovora* infection in Chinese pearleaf crab apple. Plants were inoculated with a cell suspension of *E. amylovora* (4 × 10^8^ CFU/mL) 1–2 h after treatment. Treatment with the 0.025% Tween 20 solution served as the negative control (CK), while streptomycin sulfate (100 μg/mL) was used as the positive control (PC). Disease development was assessed at 8 days after inoculation. Bars represent the mean ± standard deviation of two runs with three replicates. The different letters above the bar graphs indicate significant differences (Tukey's HSD test; *P* < 0.05). **B**–**D** Representative images of apple plants treated with crude extracts and compounds

To investigate the fire blight control efficacy, Chinese pearleaf crab apple plants were treated with two crude extracts (LE and AE) and their respective major active compounds gliotoxin (**8**) and bisdethiobis(methylthio)gliotoxin (**9**) prior to inoculation with *E. amylovora* (Fig. 5). At 8 days after inoculation, untreated plants (CK) showed severe fire blight symptoms, including necrosis of terminal buds, petioles, and leaves, while no symptoms were observed in the positive control treated with streptomycin sulfate (100 μg/mL). As shown in Fig. 5, plants treated with LE and AE extracts (500 μg/mL) displayed high levels of disease suppression, with control values of 93% and 88%, respectively. Gliotoxin (**8**) and bisdethiobis(methylthio)gliotoxin (**9**) achieved comparable disease suppression, with control values of 85% and 89% at lower concentration of 100 and 500 μg/mL, respectively. No phytotoxic effects were observed in any treated plants (Fig. 5).

## Discussion

The present study demonstrates that halogen-based OSMAC cultivation is an effective strategy for expanding the secondary metabolite repertoire and enhancing the plant disease control potential of the marine-derived fungus *T. virens* B211. Systematic supplementation with different halogen salts revealed that sodium bromide exerted a pronounced effect on both secondary metabolism and antibacterial activity. Although bromide supplementation has frequently been associated with the induction of brominated secondary metabolites in microorganisms [14, 15, 32], no new brominated compounds were detected in this study. This observation suggests that, in *T. virens* B211, bromide ions function primarily as regulatory or physiological elicitors rather than as direct halogen precursors. The elicitation effect may therefore arise from bromide-mediated modulation of secondary metabolism, enzyme activity, or stress-related signaling pathways, rather than from halogen incorporation into metabolite scaffolds. Similar uncoupling between halide supplementation and halogenated product formation has been reported in other marine-derived fungi; for example, several *Aspergillus*, *Fusarium*, and *Talaromyces* species exhibit altered or enhanced secondary metabolite profiles upon halide or salt supplementation without producing halogenated analogues, and comprehensive OSMAC-based studies have shown that chemical elicitation can activate cryptic biosynthetic pathways without necessarily yielding halogenated metabolites [33–35]. Together, these observations highlight the strain-specific nature of halide responsiveness.

Chemical investigation of NaBr-induced cultures led to the isolation of fourteen secondary metabolites, including two new viridin-family terpenoids. The predominance of viridin derivatives among the isolated compounds is consistent with previous reports on other *Trichoderma* species [9, 36], yet the discovery of new analogues underscores the capacity of OSMAC-based modulation to access previously unexplored chemical space. In particular, neoviridin (**1**) and nodulisporiviridin G (**2**), which are structural isomers, displayed clearly distinct bioactivity profiles: compound **1** exhibited antifungal activity, whereas compound **2** showed antibacterial activity. These findings reveal a pronounced structure–activity relationship within the viridin family, demonstrating that subtle stereochemical differences can result in functional divergence.

Among the viridin derivatives, viridin (**3**) and *β*-viridin (**4**) exhibited broad-spectrum antibacterial and antifungal activities, supporting their role as key contributors to the antagonistic potential of *Trichoderma* species. In contrast, norviridin (**5**) did not exhibit significant antimicrobial activity against the tested plant pathogens. Nevertheless, its isolation remains chemically noteworthy. Structurally, norviridin (**5**) represents a rare variant within the viridin family, distinguished by a modified steroid-like framework. The discovery of norviridin (**5**) under NaBr-induced OSMAC conditions highlights the ability of cultivation-based modulation to expand the chemical diversity of secondary metabolites produced by *T. virens* B211. Although norviridin (**5**) itself showed limited bioactivity, such structurally unique metabolites may serve as valuable scaffolds for future structure–activity relationship studies or chemical derivatization and contribute to a more comprehensive understanding of viridin-related biosynthetic diversity.

Gliotoxin (**8**), an epipolythiodioxopiperazine characterized by a transannular disulfide bridge, exhibited the most potent antimicrobial activity against all tested plant pathogenic bacteria and fungi, including *E. amylovora*. The substantially reduced activity of bisdethiobis(methylthio)gliotoxin (**9**), which lacks this disulfide bridge, highlights the importance of this structural feature for antimicrobial efficacy. These results are consistent with previous studies demonstrating that the redox-active disulfide bond is a key determinant of the biological activity of gliotoxin and related compounds [37, 38]. However, recent investigations have revealed that gliotoxin inhibits bacterial growth through multiple mechanisms beyond oxidative stress. These include protein thiol modification, induction of heat shock responses, protein aggregation, and disruption of metal ion homeostasis, particularly through zinc depletion [39, 40]. Collectively, these findings support a multifactorial mode of antibacterial action for gliotoxin in bacterial systems. In addition to its direct antimicrobial effects, gliotoxin has also been reported to function as a microbial signal in *Trichoderma*–plant interactions, where it primes plant immune responses and enhances systemic resistance. Comparative studies using gliotoxin-producing and nonproducing *T. virens* strains demonstrated that gliotoxin producers confer stronger protection against foliar pathogens, accompanied by the induction of defense-related genes. These observations indicate that gliotoxin contributes to disease suppression not only through direct inhibition of pathogens but also via modulation of host plant immunity [41].

At the same time, gliotoxin is well recognized for its cytotoxic effects in mammalian systems, raising concerns regarding its direct application as a standalone agrochemical. In this context, it is noteworthy that effective suppression of fire blight was achieved not only with the purified gliotoxin but also with NaBr-induced crude extracts containing multiple metabolites. The strong disease control efficacy observed for crude extracts indicates that plant disease suppression cannot be attributed solely to gliotoxin, but rather to the combined action of multiple secondary metabolites produced by *T. virens* B211. Such synergistic or additive effects are consistent with the multifactorial nature of *Trichoderma*-mediated biocontrol, where complex metabolite mixtures, together with the producing organism, contribute to disease suppression [7, 8, 10]. Indeed, crude extracts of *Trichoderma* spp. have frequently been reported to display stronger or broader antimicrobial activity than individual purified compounds, owing to the simultaneous presence of multiple bioactive metabolites such as viridins, epipolythiodioxopiperazines, and other polyketides [9, 11, 42]. In several cases, individual metabolites exhibit moderate or selective activity, whereas their combined presence results in enhanced pathogen suppression [42–45].

Consistent with previous studies, the biocontrol activity of *Trichoderma* spp. is recognized as multifactorial, involving not only direct antimicrobial effects but also mycoparasitism, enzymatic degradation of pathogens, induction of plant defense responses, and competition for nutrients and ecological niches [7, 8, 10]. Within this framework, secondary metabolites play a central role by providing chemical means of pathogen suppression while complementing other biological control mechanisms.

## Conclusion

Halogen-based OSMAC cultivation effectively expanded the secondary metabolite repertoire of *Trichoderma virens* B211, with sodium bromide exerting the most pronounced influence on both chemical diversity and biological activity. The discovery of two new viridin derivatives, together with the identification of known antifungal and antibacterial metabolites, highlights the capacity of environmental modulation to unlock novel bioactive compounds. Importantly, NaBr-induced extracts enriched in gliotoxin and bisdethiobis(methylthio)gliotoxin, as well as the purified metabolites themselves, suppressed fire blight symptoms without causing any phytotoxic effects, thereby confirming their safety for plants. The observed disease suppression appears to result from synergistic interactions among multiple metabolites rather than the action of a single compound, underscoring the ecological relevance of complex mixtures in biocontrol. Collectively, these findings demonstrate that halogen-driven OSMAC strategies provide a sustainable and safe approach to enhance the biocontrol potential of *T. virens* B211, and future studies should focus on elucidating regulatory mechanisms of metabolite biosynthesis, optimizing formulations, and validating efficacy under field conditions to fully realize their agricultural applicability.

## Supplementary Information

Supplementary material 1. Figures S1–S19 and Tables S1–S3 provides detailed isolation schemes, analytical spectra, and bioassay data supporting compound identification and activity.

## Data Availability

All data supporting the findings of this study are available within the paper and its Supplementary Information file. Raw data files in alternative formats are available from the corresponding author upon reasonable request.
