# Supplementary material for: Metabolite diversification via halogen salts enhances biocontrol potential of marine-derived Trichoderma virens B211
Source: Nat Prod Bioprospect. 2026 Jun 23;16(1):72. doi: 10.1007/s13659-026-00634-y (PMC13291281; doi:10.1007/s13659-026-00634-y)
Supplement: Supplementary file 1 — Supplementary material 1. Figures S1–S19 and Tables S1–S3 provides detailed isolation schemes, analytical spectra, and bioassay data supporting compound identification and activity. [file 13659_2026_634_MOESM1_ESM.docx]

**Supplementary Materials**

**Metabolite diversification via halogen salts enhances biocontrol potential of marine-derived *Trichoderma virens* B211**

Minh Van Nguyen^1^, Yeong Seok Kim^1^, Seung Mok Ryu^2^, Jae Young Kwon^3^, Hun Kim^1,4^, Gyung Ja Choi^1,4*^, Jae Woo Han^1,4*^

^1^Eco-Friendly New Materials Research Center, Korea Research Institute of Chemical Technology, Daejeon 34114, Republic of Korea

^2^Herbal Medicine Resources Research Center, Korea Institute of Oriental Medicine, Naju 58245, Republic of Korea

^3^KIST Gangneung Institute of Natural Products, Korea Institute of Science and Technology, Gangneung 25451, Republic of Korea

^4^Department of Medicinal Chemistry and Pharmacology, University Science and Technology, Daejeon 34113, Republic of Korea.

^*^Correspondance:

Gyung Ja Choi

ORCID: [0000-0003-1564-1487](https://orcid.org/0000-0003-1564-1487)

E-mail: [kjchoi@krict.re.kr](mailto:kjchoi@krict.re.kr)

Jae Woo Han

ORCID: [0000-0003-1165-8433](https://orcid.org/0000-0003-1165-8433)

E-mail: [jaewoo82@krict.re.kr](mailto:jaewoo82@krict.re.kr)

**Table of contents**

**Figure S1.** Isolation scheme of compounds **1–14** from the marine-derived *Trichoderma virens* B11. [3](#FigS1)

**Figure S2.** HPLC profiles of ethyl acetate extracts of *Trichoderma virens* B211 cultured in 50 mL potato dextrose broth supplemented with 0%, 1%, 3%, and 6% NaBr (w/v). [4](#FigS2)

**Figure S3.** HPLC profiles of ethyl acetate extracts, LE and AE, from liquid and solid cultures of the *Trichoderma virens* B211.. [5](#FigS3)

**Figure S4.** High resolution mass spectrometry of compound **1** obtained in electrospray ionization positive mode. [6](#FigS4)

**Figure S5**. ^1^H NMR spectrum of compound **1** (400 MHz, CDCl_3_). [7](#FigS5)

**Figure S6.** ^13^C NMR spectrum of compound **1** (100 MHz, CDCl_3_). [8](#FigS6)

**Figure S7.** COSY spectrum of compound **1** (400 MHz, CDCl_3_). [9](#FigS7)

**Figure S8.** HSQC spectrum of compound **1** (400 MHz, CDCl_3_). [10](#FigS8)

**Figure S9**. HMBC spectrum of compound **1** (400 MHz, CDCl_3_). [11](#FigS9)

**Figure S10.** 1D NOESY spectra of the compounds **1** and **2** (A and B) (500 MHz, CDCl_3_). [12](#FigS10)

**Figure S11.** High resolution mass spectrometry of compound **5** obtained in electrospray ionization positive mode. [13](#FigS11)

**Figure S12.** ^1^H spectrum of compound **5** (400 MHz, DMSO-*d*_6_). [14](#FigS12)

**Figure S13.** ^13^C NMR spectrum of compound **5** (400 MHz, DMSO-*d*_6_). [15](#FigS13)

**Figure S14.** COSY spectrum of compound **5** (400 MHz, DMSO-*d*_6_). [16](#FigS14)

**Figure S15.** HSQC spectrum of compound **5** (400 MHz, DMSO-*d*_6_). [17](#FigS15)

**Figure S16.** HMBC spectrum of compound **5** (400 MHz, DMSO-*d*_6_). [18](#FigS16)

**Figure S17.** Comparison between the experimental and calculated ECD spectra of (3*R*,4*R*,8*R*)-, (3*S*,4*S*,8*S*)-, (3*R*,4*R*,8*S*)-, (3*S*,4*S*,8*R*)-, (3*R*,4*S*,8*R*)-, and (3*S*,4*R*,8*S*)-stereoisomers of compounds **5**. [19](#FigS17)

**Figure S18.** Low-resolution electrospray ionization mass spectrum (ESI-MS, positive mode) of compound **11**. [20](#FigS18)

**Figure S19.** ^1^H, ^13^C, and DEPT135 spectra (A and B) of compound **11** measured in DMSO-*d*_6_ at 400 MHz. [21](#FigS19)

**Table S1**. List of plant pathogenic bacteria and fungi used in this study. [22](#TableS1)

**Table S2**. Minimum inhibitory concentrations (MICs) of ethyl acetate extracts from *Trichoderma virens* B211 cultured in potato dextrose broth (PDB) supplemented with 1%, 3%, and 6% of NaBr against *Erwinia amylovora* TS3128. [23](#TableS2)

**Table S3**. NMR and MS spectroscopic data of known compounds **2**–**4** and **6**–**14** isolated from *Trichoderma virens* B211. [24](#TableS3)

**Table S4**. DP4+ probability analysis of the two stereoisomers of compound **5** based on experimental and calculated NMR chemical shifts (B3LYP/6‑311+G(d,p), PCM in DMSO), using scaled (sDP4+), unscaled (uDP4+), and original DP4+ methods with proton (^1^H), 13-carbon (^13^C), and combined (all) data. [26](#TableS4)

**Table S5**. ^13^C NMR data of compound **11**. [27](#TableS5)


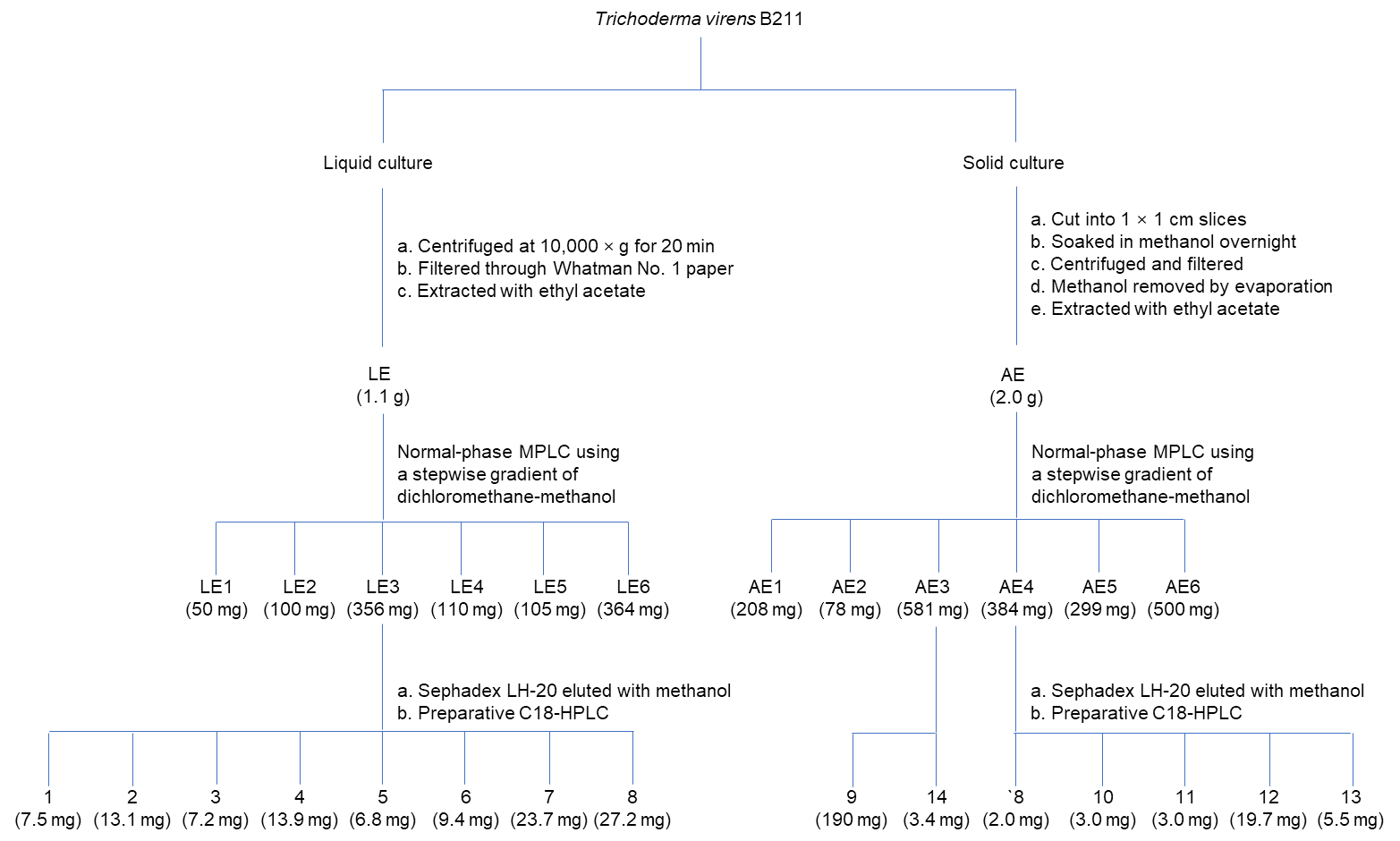


**Fig**. **S1** Isolation scheme of compounds **1–14** from the marine-derived *Trichoderma virens* B211. Mycelial disks (5 mm in diameter) were used as inoculum. Liquid culture was carried out in 8 L of potato dextrose broth supplemented with 3% NaBr (w/v) at 25ºC, 160 r/min for 14 days. Solid culture was performed on 6 kg of potato dextrose agar plates (150 mm in diameter) at 25ºC for 14 days. Two types of ethyl acetate extracts from liquid and solid cultures were filtered, dried, and designated as LE and AE, respectively


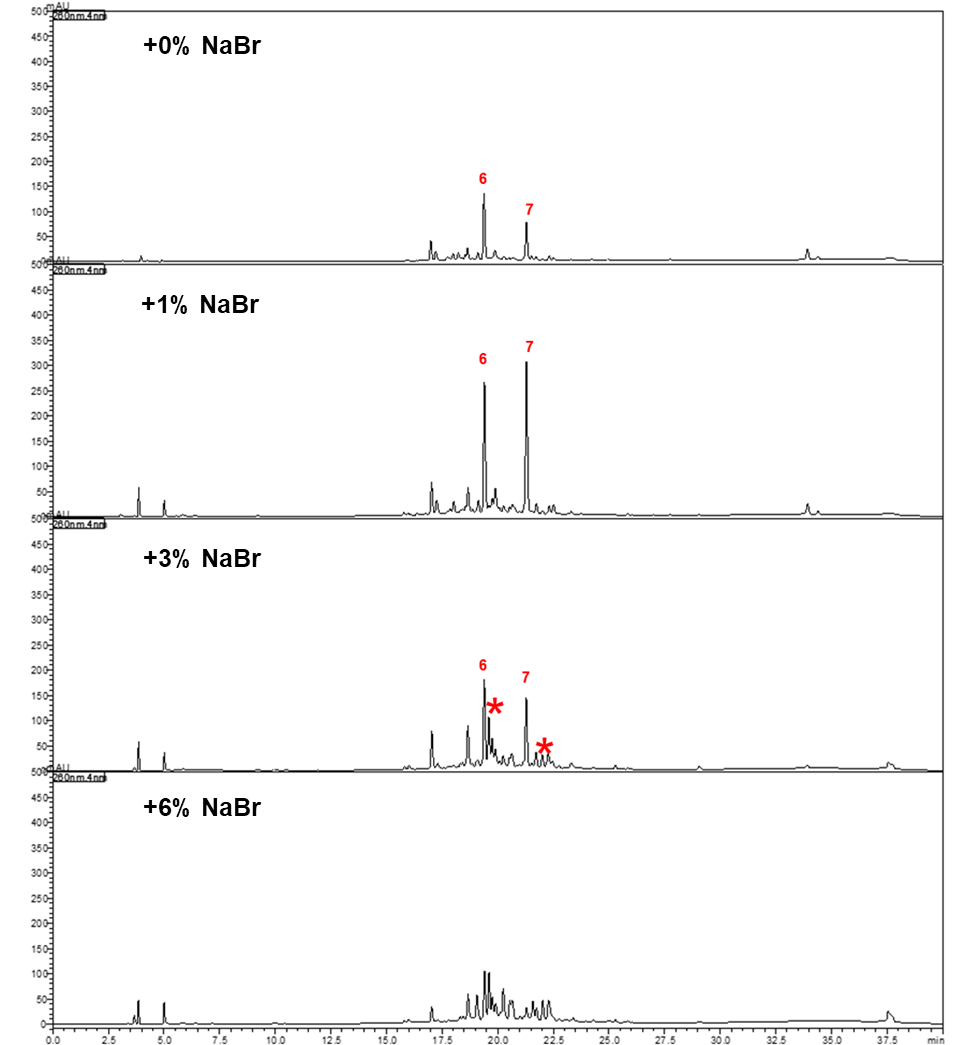


**Fig**. **S2** HPLC profiles of ethyl acetate extracts of Trichoderma virens B211 cultured in 50 mL potato dextrose broth supplemented with 0%, 1%, 3%, and 6% NaBr (w/v). Analyses were performed on a Shimadzu LC-20AR system equipped with a Phenomenex Luna C18(2) column (250 x 4.6 mm, 5 μm) at a flow rate of 0.8 mL/min. Solvent system: A, water; B, acetonitrile. Elution program: 0–5min, isocratic 10% B; 5–25 min, gradient 10–100% B; 25–30 min, isocratic 100% B; 30–32min, 100–10% B; 32–40min, isocratic 10% B. 1β-hydroxyl-2α-hydroasterogynin A (**6**) and asterogynin A (**7**)


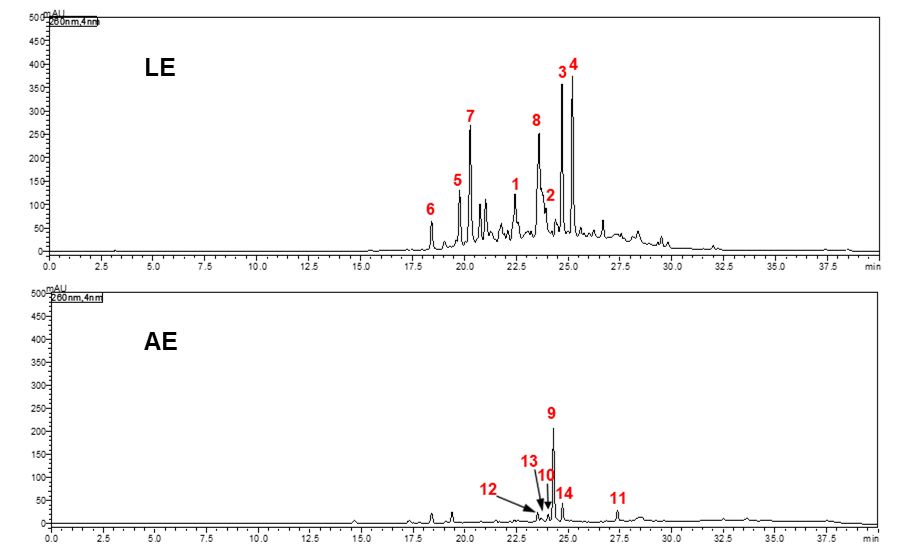


**Fig**. **S3** HPLC profiles of ethyl acetate extracts, LE and AE, from liquid and solid cultures of the Trichoderma virens B211. Analyses were conducted on a Shimadzu LC-20AR system equipped with a Phenomenex Luna C18(2) column (250 x 4.6 mm, 5 μm) at a flow rate of 1 mL/min. Solvent system: A, water; B, acetonitrile. Elution program: 0–5min, isocratic 20% B; 5–25 min, gradient 20–100% B; 25–30 min, isocratic 100% B; 30–32min, 100–20% B; 32–40min, isocratic 20% B. Identified compounds: isocratic 20% B. nodulisporiviridin I (**1**), nodulisporiviridin G (**2**), α-viridin (**3**), β-viridin (**4**), nodulisporiviridin J (**5**), 1β-hydroxyl-2α-hydroasterogynin A (**6**), asterogynin A (**7**), gliotoxin (**8**), bisdethiobis(methylthio)gliotoxin (**9**), (S)-3-(hydroxymethyl)-2-methyl-2,3-dihydropyrazino[1,2-a]indole-1,4-dione (**10**), 3-(hydroxymethyl)-2-methyl-2,3-dihydropyrazino[1,2-a]indole-1,4-dione (**11**), hydroheptelidic acid (**12**), xylaric acid B (**13**), and 5-hydroxyl-3-hydroxymethyl-2-methyl-7-methoxychromone (**14**)


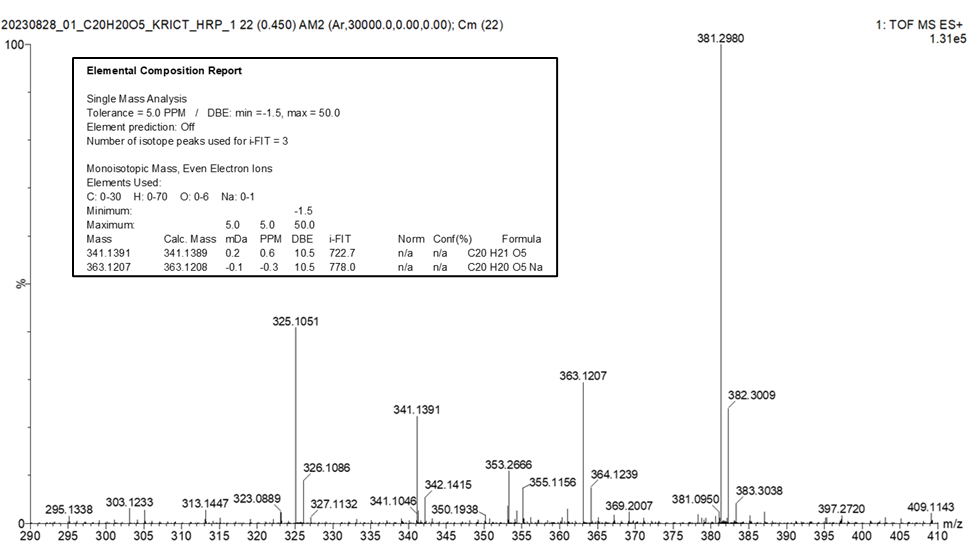


**Fig**. **S4** High resolution mass spectrometry of compound **1** obtained in electrospray ionization positive mode


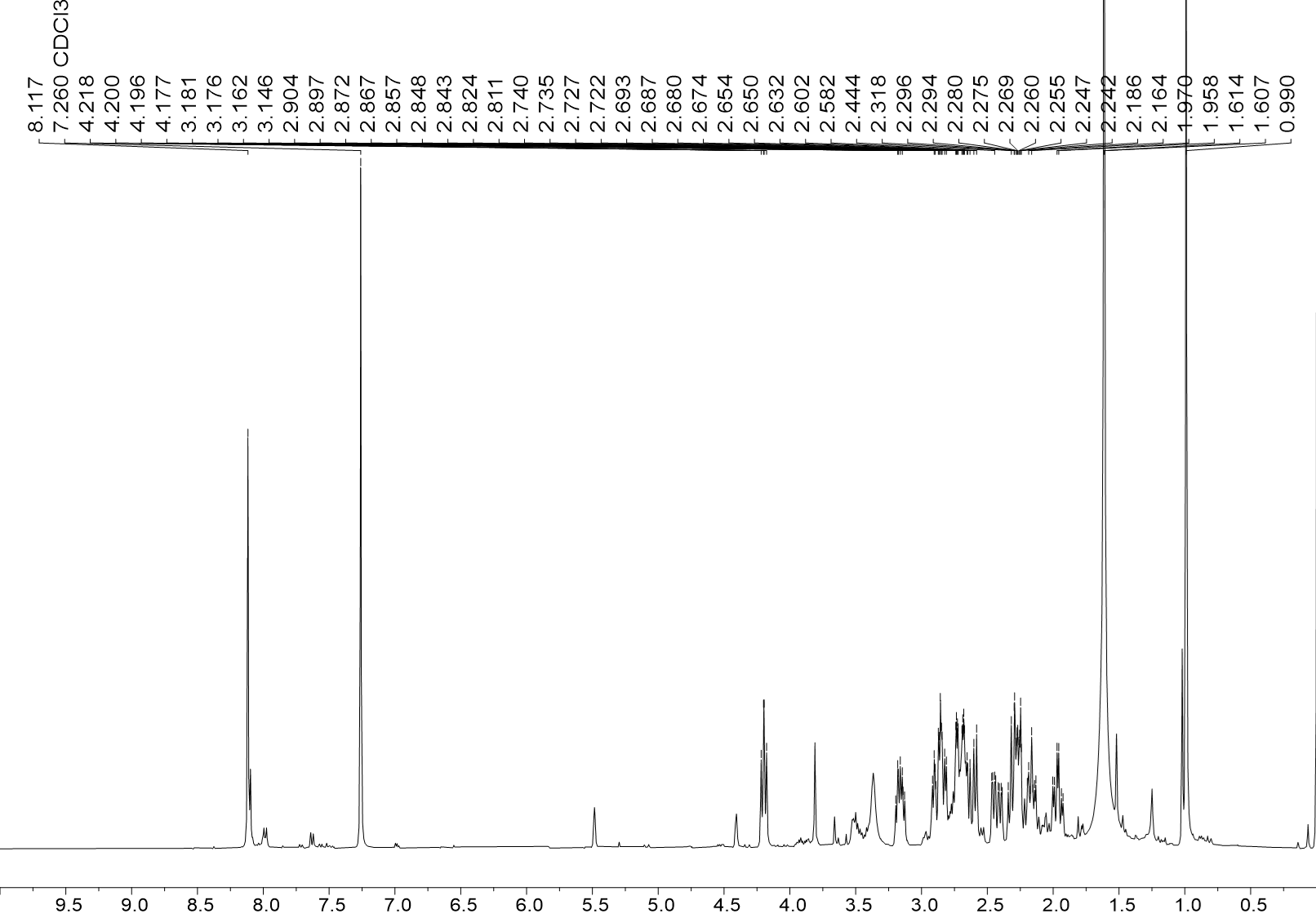


**Fig**. **S5** ^1^H NMR spectrum of compound **1** (400 MHz, CDCl_3_)


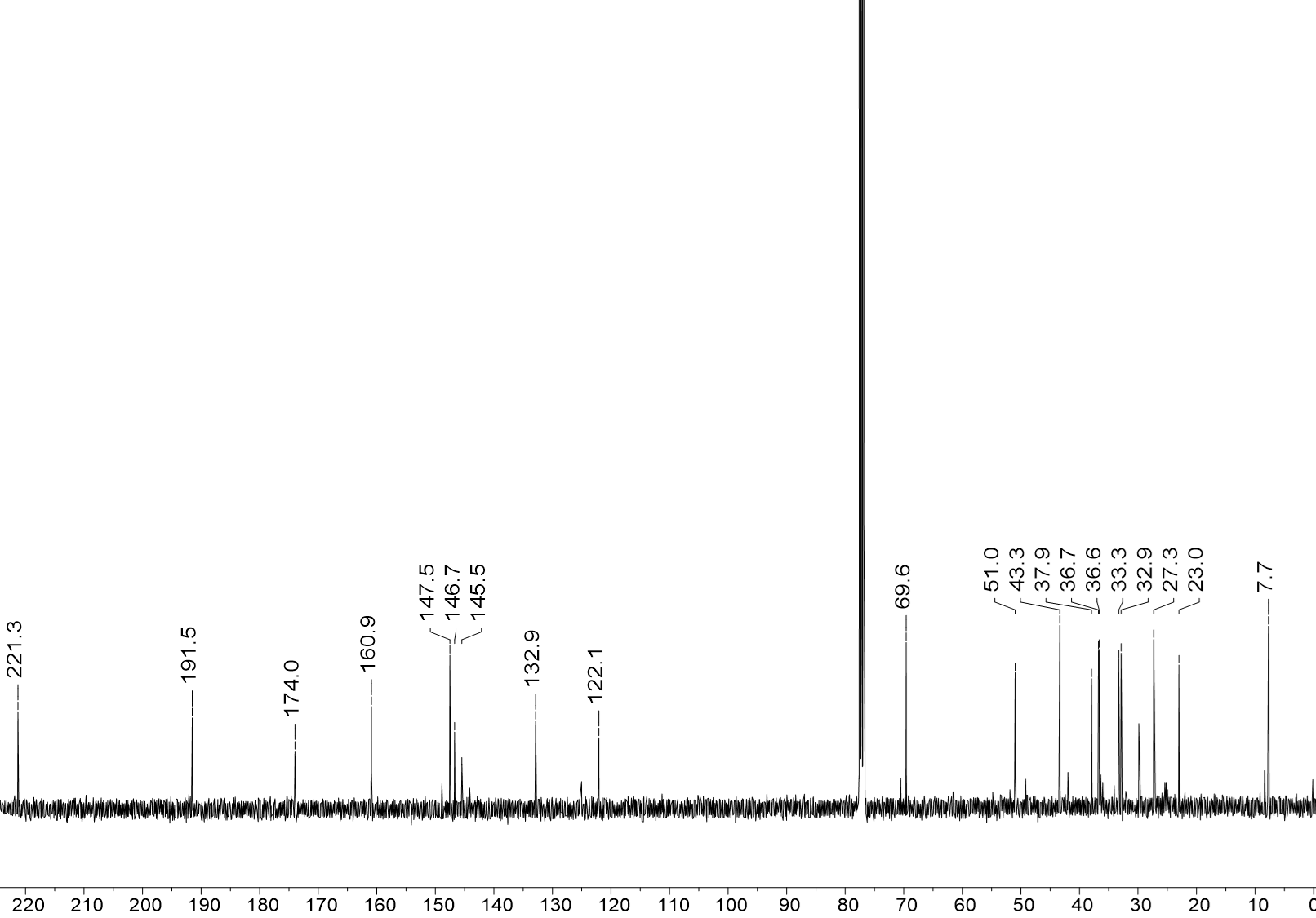


**Fig**. **S6** ^13^C NMR spectrum of compound **1** (100 MHz, CDCl_3_)

**Fig**. **S7** COSY spectrum of compound **1** (400 MHz, CDCl_3_)

**Fig**. **S8** HSQC spectrum of compound **1** (400 MHz, CDCl_3_)

**Fig**. **S9** HMBC spectrum of compound **1** (400 MHz, CDCl_3_)


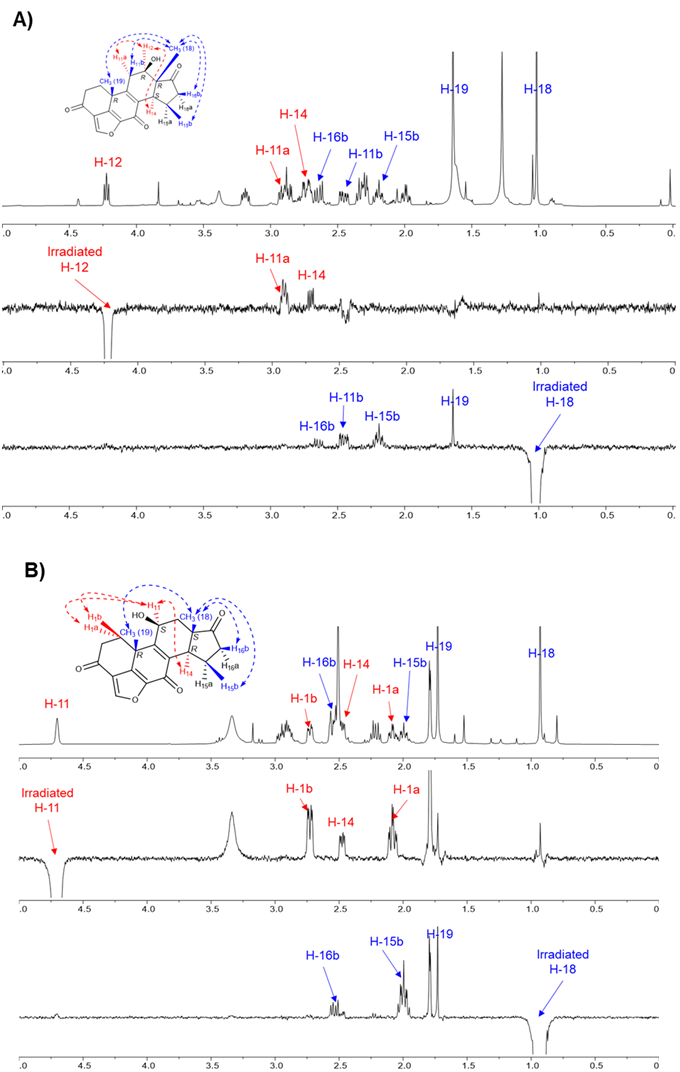


**Fig**. **S10** 1D NOESY spectra of the compounds **1** and **2** (A and B) (500 MHz, CDCl_3_)

**Fig**. **S1**1 High resolution mass spectrometry of compound **5** obtained in electrospray ionization positive mode


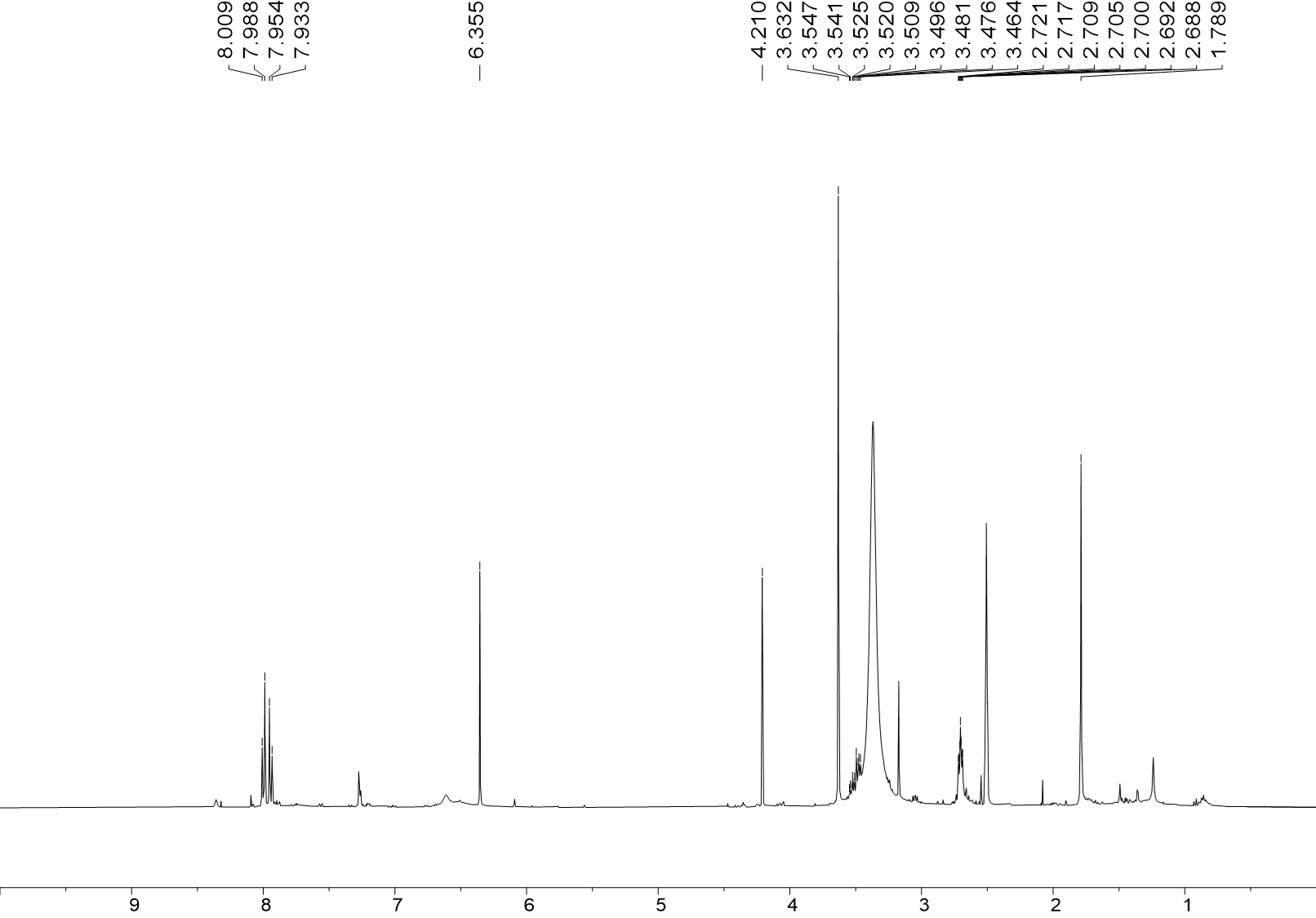


**Fig**. **S12** ^1^H spectrum of compound **5** (400 MHz, DMSO-d_6_)

**Fig**. **S13** ^13^C NMR spectrum of compound **5** (400 MHz, DMSO-d_6_)

**Fig**. **S14** COSY spectrum of compound **5** (400 MHz, DMSO-d_6_)

**Fig**. **S15** HSQC spectrum of compound **5** (400 MHz, DMSO-d_6_)

**Fig**. **S16** HMBC spectrum of compound **5** (400 MHz, DMSO-d_6_)


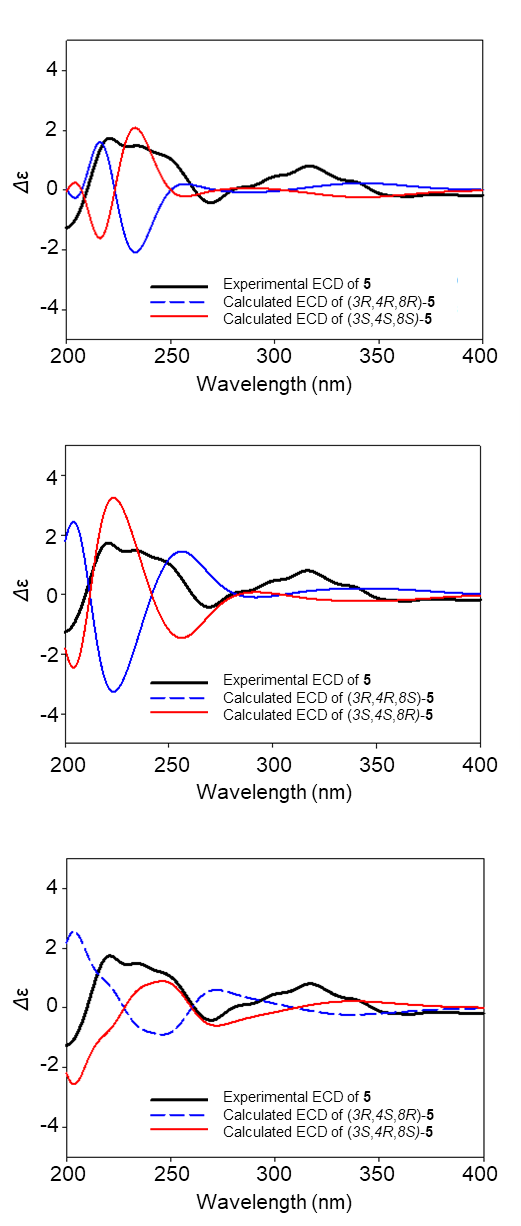


**Fig**. **S17** Comparison between the experimental and calculated ECD spectra of (3*R*,4*R*,8*R*)-, (3*S*,4*S*,8*S*)-, (3*R*,4*R*,8*S*)-, (3*S*,4*S*,8*R*)-, (3*R*,4*S*,8*R*)-, and (3*S*,4*R*,8*S*)-stereoisomers of compounds **5**. The experimental spectra are shown as a solid black line, while the calculated spectra (TD-DFT, B3LYP/6-31G*) are displayed as dashed blue or solid red lines

**Fig**. **S18** Low-resolution electrospray ionization mass spectrum (positive mode) of compound **11**


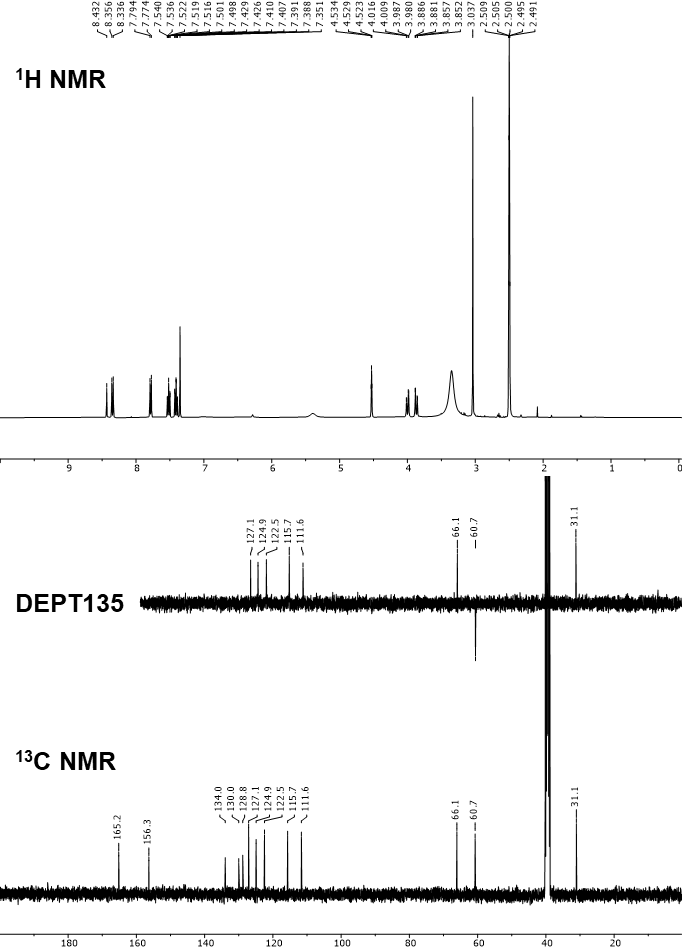


**Fig**. **S19** ^1^H, ^13^C, and DEPT135 spectra of compound **11** measured in DMSO-d_6_ at 400 MHz

**Table S1** List of plant pathogenic bacteria and fungi in this study

| **Scientific name** | **Strain** | **Plant disease** | **Ref.^a^** |
| --- | --- | --- | --- |
| ***Bacteria*** |  |  |  |
| *Acidovorax citrulli* | KACC 17005 | Bacterial fruit blotch of watermelon and melon | [1] |
| *Agrobacterium tumefaciens* | SL2434 | Crown gall | [1] |
| *Burkholderia glumae* | SL4269 | Bacterial grain rot | [1] |
| *Dickeya chrysanthemi* | SL3218 | Soft rot | [1] |
| *Erwinia amylovora* | TS3128 | Fire blight of apple and pear | [2] |
| *Pectobacterium carotovorum subsp. carotovorum* | SL290 | Soft rot | [1] |
| *Pseudomonas syringae* pv. *actinidiae* | CJW7 | Bacterial canker of kiwifruit | [1] |
| *Pseudomonas syringae* pv. *lachrymans* | SL308 | Angular leaf spot of cucumber | [1] |
| *Ralstonia solanacearum* | SL1944 | Bacterial wilt of tomato | [1] |
| *Xanthomonas arboricola* pv. *pruni* | SL4370 | Bacterial shot-hole of peach | [1] |
| *Xanthomonas campestris* pv. *campestris* | KACC 10377 | Black rot of cabbage and Chinese cabbage | [1] |
| ***Fungi*** |  |  |  |
| *Alternaria brassicicola* | KACC 40036 | Black spot of radish and Chinese cabbage | [3],[4] |
| *Alternaria porri* | KACC 42998 | Alternaria leaf spot/Purple blotch | [3],[4] |
| *Botrytis cinerea* | KACC 48736 | Gray mold | [3],[4] |
| *Cladosporum cucumerinum* | KACC 40576 | Scab of cucurbits | [4] |
| *Colletotrichum coccodes* | KACC 48737 | Chili pepper anthracnose | [3],[4] |
| *Cylindrocarpon destructans* | KACC 41077 | Root rot of ginseng | [4] |
| *Fusarium oxysporum* | KACC 40043 | Fusarium wilt | [3],[4] |
| *Fusarium fujikuroi* | KACC 46888 | Bakanae disease | [5] |
| *Fusarium graminearum* | Z-3639 | Fusarium head blight | [6] |
| *Magnaporthe oryzae* | KACC 46552 | Rice blast | [3],[4] |
| *Phytophthora infestans* | KACC 48738 | Late blight of potato/tomato | [3],[4] |
| *Stemphylium vesicarium* | KACC 44530 | Leaf blight of onion/garlic | [7] |

^a^References:

1. T. T. Vu, H, Kim, V. K. Tran, H. D. Vu, T. X. Hoang, J. W. Han, Y. H. Choi, K. S. Jang, G. J. Choi, J. C. Kim, Antibacterial activity of tannins isolated from *Sapium baccatum* extract and use for control of tomato bacterial wilt, PloS One 12 (7) (2017) e0181499.
2. Y. S. Kim, E. J. Kang, D. Kim, B. Kim, J. W. Han, J. H. Lee, S. W. Han, G. J. Choi, H. Kim, Tizoxanide associated with anti-virulent activity controls fire blight disease caused by *Erwinia amylovora* in *Malus asiatica*, Commun. Biol. 8 (1) (2025) 1451.
3. M. Y. Kim, J. W. Han, Q. L. Dang, J. C. Kim, H. Kim, G. J. Choi, Characterization of *Alternaria porri* causing onion purple blotch and its antifungal compound magnolol identified from *Caryodaphnopsis baviensis*. PloS One, 17 (1) (2022) e0262836
4. J. H. Park, J. W. Han, B. Kim, S. U. Park, G. J. Choi, H. Kim, Suppression of rice blast and tomato late blight by *Paraboeremia adianticola* producing vulculic acid, Pest Manag. Sci. 81 (9) (2025) 5052–5061
5. S. Shin, J. W. Lee, N. Lee, J. W. Han, G. J. Choi, H. Son, H. Kim, Genome sequence data of *Pseudalkalibacillus hwajinpoensis* MABIK MI00000821, an Inhibitor of fumonisin production in *Fusarium fujikuroi*, PhytoFront. 4 (4) (2024), 821–824.
6. J. Park, J. W. Han, N. Lee, S. Kim, S. Choi, H. H. Lee, J. E. Kim, Y. S. Seo, G. J. Choi, Y. W. Lee, H. Kim, H. Son, Sulfur metabolism-mediated fungal glutathione biosynthesis is essential for oxidative stress resistance and pathogenicity in the plant pathogenic fungus Fusarium graminearum, Mbio 15 (1) (2024) e02401-23.
7. J. J. Lee, J. W. Han, H. Kim, J. C. Kim, G. J. Choi, Evaluation of onion germplasm for resistance to leaf blight caused by *Stemphylium vesicarium*, Res. Plant Dis. 30 (4) (2024) 342–352.

**Table S2** Minimum inhibitory concentrations (MICs) of ethyl acetate extracts from *Trichoderma virens* B211 cultured in potato dextrose broth (PDB) supplemented with 1%, 3%, and 6% of NaBr against *Erwinia amylovora* TS3128

| **NaBr (%)** | **MIC values (μg/mL) of**  **ethyl acetate extracts** |
| --- | --- |
| 0 | 25 |
| 1 | 25 |
| 3 | 12.5 |
| 6 | 50 |

**Table S3** NMR and MS spectroscopic data of known compounds **2–4** and **6–14** isolated from Trichoderma virens B211

| Compound | Solvent | *δ*_H_ (mult, *J*, Int.)^a^ | *δ*_C_ | ESIMS (m/z) | Ref.^b^ |
| --- | --- | --- | --- | --- | --- |
| Nodulisporiviridin G (**2**) | DMSO-*d*_6_ | 8.65 (s, 1H), 5.63 (br s, 1H), 4.71 (br s, 1H), 3.00–2.86 (m, 2H), 2.73 (ddd, 13.1, 5.4, 2.1, 1H), 2.58–2.56 (m, 1H), 2.49–2.45 (m, 1H), 2.26–2.17 (m, 1H), 2.12–2.04 (m, 1H), 1.99 (ddd, 12.8, 9.1, 3.9, 1H), 1.79 (d, 3.9, 2H), 1.73 (s, 3H), 0.93 (s. 3H) | 217.9, 192.6, 174.6, 163.9, 149.1, 149.0, 144.4, 132.6, 122.1, 67.1, 46.8, 44.4, 38.6, 36.8, 36.7, 30.9, 29.0, 23.2, 14.8 | 341 [M + H]^+^ | [1] |
| Viridin (**3**) | CDCl_3_ | 8.64 (d, 8.2, 1H), 8.30 (s, 1H), 7.98 (d, 8.2, 1H), 4.28 (d, 9.5, 1H), 4.17 (d, 9.5, 1H), 3.89 (s, 3H), 3.80 (t, 5.8, 1H), 3.70 (t, 5.8, 1H), 3.39, (br s, 1H), 2.77–2.74 (m, 2H), 1.74 (s, 3H) | 206.6, 189.2, 173.4, 158.2, 155.8, 148.7, 146.9, 142.2, 137.5, 129.9, 128.5, 127.8, 122.3, 86.0, 75.6, 61.9, 41.9, 36.5, 28.6, 27.4 | 353 [M + H]^+^ | [2] |
| *β*-Viridin (**4**) | CDCl_3_ | 8.65 (d, 8.2, 1H), 8.33 (s, 1H), 7.98 (d, 8.2, 1H), 4.39–4.34 (m, 1H), 3.93–3.85 (m, 1H), 3.85–3.78 (m, 1H), 3.75 (s, 3H), 3.71–3.64 (m, 1H), 2.77–2.74 (m, 2H), 1.68 (s, 3H) | 206.6, 187.1, 173.4, 158.2, 156.6, 149.5, 147.1, 143.2, 137.3, 129.7, 128.0, 127.8, 121.8, 83.6, 73.4, 61.1, 42.1, 36.5, 29.9, 28.6 | 353 [M + H]^+^ | [2] |
| 1*β*-Hydroxyl-2*α*-hydroasterogynin A (**6**) | CD_3_OD | 8.06 (d, 8.0, 1H), 7.79 (d, 8.0, 1H), 4.45 (d, 2.2, 1H), 3.44 (dd, 6.9, 4.7, 2H), 3.41 (dd, 2.2, 0.7, 1H), 3.27 (s, 3H), 2.93 (dd, 17.1, 0.8, 1H), 2.80 (d, 17.1, 1H), 2.78–2.76 (m, 1H), 1.52 (s, 3H) | 207.7, 206.2, 205.8, 166.1, 156.9, 139.2, 131.7, 131.5, 124.9, 84.6, 80.9, 76.7, 58.3, 53.1, 46.7, 37.2, 25.9, 21.5 | 331 [M + H]^+^, 353 [M + Na]^+^ | [3] |
| Asterogynin A (**7**) | CD_3_OD | 7.93 (d, 7.6, 1H), 7.71 (d, 7.8, 1H), 5.98 (br s, 1H), 3.52 (s, 3H), 3.23–3.21 (m, 3H), 2.87 (dd, 16.5, 4.6, 1H), 2.68–2.64 (m, 3H), 1.47 (br s, 3H) | 204.7, 201.1, 189.3, 163.0, 153.7, 147.4, 135.9, 128.6, 127.5, 121.7, 117.6, 79.4, 52.8, 41.7, 34.3, 22.9, 22.1 | 335 [M + Na]^+^ | [4] |
| Gliotoxin (**8**) | CDCl_3_ | 6.00 (m, 1H), 5.94 (dd, 9.4, 4.7, 1H), 5.78 (d, 9.5, 1H), 5.62 (s, 1H), 4.81 (s, 2H), 4.41 (dd, 12.8, 5.9, 1H), 4.26 (dd, 12.9, 9.8, 1H), 3.74 (dd, 18.1, 1.7, 1H), 3.52 (dd, 9.8, 5.9, 1H), 3.20 (s, 3H), 2.95 (d, 17.9, 1H) | 166.2, 165.5, 130.8, 130.2, 123.5, 120.4, 77.4, 77.3, 73.3, 70.0, 60.8, 36.7, 27.6 | 327 [M + H]^+^ | [5] |
| Bisdethiobis(methylthio)gliotoxin (**9**) | DMSO-*d*_6_ | 5.99 (m, 1H), 5.93 (m, 1H), 5.68 (m, 1H), 4.92 (br s, 1H), 4.88 (br s, 1H), 4.26 (d, 11.5, 1H), 3.87 (d, 11.5, 1H), 3.12 (s, 3H), 3.11 (m, 1H), 2.94 (dd, 15.8, 2.6, 1H), 2.27 (s, 3H), 2.25 (s, 3H) | 168.4, 167.8, 134.0, 130.8, 124.8, 120.8, 75.7, 74.4, 73.2, 70.5, 64.7, 39.7, 29.1, 15.2, 13.5 | 379 [M + Na]^+^ | [6] |
| (3*S*)-2,3-Dihydro-6-hydroxy-3-(hydroxymethyl)-2-methylpyrazino[1,2-*α*]indole-1,4-dione (**10**) | DMSO-*d*_6_ | 7.78 (d, 8.0, 1H), 7.34 (d, 1.0, 1H), 7.31 (d, 8.0, 1H), 6.76 (dd, 8.0, 1.0, 1H), 4.49 (t, 2.0, 1H), 3.98 (dd, 12.0, 3.0, 1H), 3.85 (dd, 12.0, 2.0, 1H), 3.02 (s, 3H) | 165.1, 156.4, 151.8, 135.5, 128.6, 128.1, 118.2, 109.9, 109.1, 106.8, 66.1, 60.7, 31.0 | 261 [M + H]^+^ | [7] |
| (±)-2,3-Dihydro-3-(hydroxymethyl)-2-methylpyrazino[1,2-*α*]indole-1,4-dione (**11**) | DMSO-*d*_6_ | 8.35 (d, 8.3, 1H), 7.78 (d, 7.7, 1H), 7.52 (ddd, 8.3, 7.2, 1.3, 1H), 7.41 (td, 7.7, 1.3, 1H), 7.35 (s, 1H), 4.53 (t, 2.3, 1H), 4.00 (dd, 11.6, 2.8, 1H), 3.87 (dd, 11.6, 2.0, 1H), 3.04 (s, 3H) | 165.2, 156.3, 134.0, 130.0, 128.8, 127.1, 124.9, 122.5, 115.7, 111.6, 66.1, 60.7, 31.1 | 245 [M + H]^+^ | [8] |
| Hydroheptelidic acid (**12**) | CD_3_OD | 6.62 (d, 10.6, 1H), 4.46 (d, 9.9, 1H), 4.35 (d, 12.3, 1H), 4.11 (d, 12.3, 1H), 4.06 (dd, 9.9, 0.7, 1H), 2.67 (m, 1H), 2.11 (d, 11.0, 1H), 2.01 (dt, 13.7, 3.4, 1H), 1.81–1.74 (m, 2H), 1.67 (ddd, 13.9, 12.7, 4.6, 1H), 1.36–1.20 (m, 2H), 0.97 (d, 7.0, 3H), 0.77 (d, 6.8, 3H) | 179.4, 170.2, 145.4, 135.0, 76.4, 76.3, 57.5, 53.9, 46.6, 41.6, 32.9, 29.4, 22.0, 21.9, 15.8 | 299 [M + H]^+^ | [9] |
| Xylaric acid B (**13**) | CD_3_OD | 6.48 (d, 11.0, 1H), 4.32 (br s, 2H), 3.72 (d, 11.0, 1H), 3.47 (d, 11.0, 1H), 3.38 (s, 3H), 2.92 (q, 11.0, 1H), 2.46 (d, 11.0, 1H), 2.31 (m, 1H), 1.29 (m, 1H), 1.71 (pd, 7.0, 2.0, 1H), 1.62 (m, 1H), 1.29 (m, 1H), 1.29 (m, 1H), 0.93 (d, 7.0, 3H), 0.80 (d, 7.0, 3H) | 176.5, 170.5, 147.3, 134.6, 75.2, 73.8, 59.7, 59.5, 57.9, 47.7, 41.7, 35.9, 29.5, 21.9, 21.6, 16.1 | 353 [M + Na]^+^, 329 [M - H]^-^ | [10] |
| 5-Hydroxyl-3-hydroxymethyl-2-methyl-7-methoxychromone (**14**) | CD_3_OD | 6.48 (d, 2.2, 1H), 6.32 (d, 2.2, 1H), 4.55 (s, 2H), 3.86 (s, 3H), 2.50 (s, 3H) | 182.6, 168.5, 167.2, 163.2, 159.2, 119.7, 105.9, 98.9, 93.1, 56.4, 54.5, 18.1 | 237 [M + H]^+^ | [11] |

^a^Chemical shifts (***δ***) are reported in ppm, coupling constants (*J*) in Hz, and Int. denotes integration.

^b^References:

1. Q. Zhao, G. D. Chen, X. L. Feng, Y. Yu, R. R. He, X. X. Li, Y. Huang, W. X. Zhou, L. D. Guo, Y. Z. Zheng, Nodulisporiviridins A–H, bioactive viridins from *Nodulisporium* sp., J. Nat. Prod. 78 (6) (2015) 1221–1230.
2. A. G. Ayent, J. R. Hanson, A. Truneh, Metabolites of *Gliocladium flavofuscum*, Phytochemistry, 32(1) (1992) 197–198.
3. P. F. Andersson, S. Bengtsson, J. Stenlid, A. Broberg, B-norsteroids from *Hymenoscyphus pseudoalbidus*, Molecules, 17 (7) (2012) 7769–7781.
4. S. Cao, L. Ross, G. Tamayo, J. Clardy, Asterogynins: secondary metabolites from a Costa Rican endophytic fungus, Org. Lett. 12 (20) (2010) 4661–4663.
5. K. S. Svahn, U. Göransson, E. Chryssanthou, B. Olsen, J. Sjölin, A. A. Strömstedt, Induction of gliotoxin secretion in *Aspergillus fumigatus* by bacteria-associated molecules, Plos One 9 (4) (2014) e93685.
6. G. W. Kirby, D. J. Robins, M. A. Sefton, R. R. Talekar, Biosynthesis of bisdethiobis (methylthio) gliotoxin, a new metabolite of *Gliocladium deliquescens*, J. Chem. Soc., Perkin Trans. 1 (1980) 119–121.
7. R. R. Forseth, E. M. Fox, D. Chung, B. J. Howlett, N. P. Keller, F. C. Schroeder, Identification of cryptic products of the gliotoxin gene cluster using NMR-based comparative metabolomics and a model for gliotoxin biosynthesis, J. Am. Chem. Soc. 133 (25) (2011), 9678–9681.
8. N. R. Koning, D. Strand, AC–H activation approach to the tricyclic core of glionitrin A and B, ACS Omega 7 (14) (2022) 12329–12341.
9. M. Lee, J. Y. Cho, Y. G. Lee, H. J. Lee, S. I. Lim, S. Y. Lee, Y. D. Nam, J. H. Moon, Furan, phenolic, and heptelidic acid derivatives produced by *Aspergillus oryzae*, Food Sci. Biotechnol. 25 (5) (2016) 1259–1264.
10. S. Yan, S. Li, W. Wu, F. Zhao, L. Bao, R. Ding, H. Gao, H. A. Wen, F. Song, H. W. Liu, Terpenoid and phenolic metabolites from the fungus *Xylaria* sp. associated with termite nests. Chem. Biodivers. 8 (9) (2011) 1689–1700.
11. S. Kamisuki, C. Ishimaru, K. Onoda, I. Kuriyama, N. Ida, F. Sugawara, H. Yoshida, Y. Mizushina, Nodulisporol and nodulisporone, novel specific inhibitors of human DNA polymerase *λ* from a fungus, *Nodulisporium* sp. Bioorg. Med. Chem. 15 (9) (2007) 3109–3114.

**Table S4** DP4+ probability analysis of the two stereoisomers of compound **5** based on experimental and calculated NMR chemical shifts (B3LYP/6‑311+G(d,p), PCM in DMSO), using scaled (sDP4+), unscaled (uDP4+), and original DP4+ methods with proton (^1^H), 13-carbon (^13^C), and combined (all) data

| **Method (data type)** | **(*3S*,*4R*,*8R*)-5 (%)** | **(*3S*,*4R*,*8S*)-5 (%)** |
| --- | --- | --- |
| sDP4+ (^1^H data) | 89.88 | 10.12 |
| sDP4+ (^13^C data) | 99.99 | 0.01 |
| sDP4+ (all data) | 100 | 0 |
| uDP4+ (^1^H data) | 97.17 | 2.83 |
| uDP4+ (^13^C data) | 100 | 0 |
| uDP4+ (all data) | 100 | 0 |
| DP4+ (^1^H data) | 99.67 | 0.33 |
| DP4+ (^13^C data) | 100 | 0 |
| DP4+ (all data) | 100 | 0 |

**Table S5** ^13^C NMR data of compound **11**

| ***δ*_C_ in ppm** | |
| --- | --- |
| **In this study** | **Ref.^a^** |
| 165.2 | 165.1 |
| 156.3 | 156.3 |
| 134.0 | 134.0 |
| 130.0 | 130.0 |
| 128.8 | 128.8 |
| 127.1 | 127.1 |
| 124.9 | 124.9 |
| 122.5 | 122.5 |
| 115.7 | 115.7 |
| 111.6 | 111.6 |
| 66.1 | 66.1 |
| 60.7 | 60.7 |
| 31.1 | 31.1 |
| **^a^**Reference: N. R. Koning, D. Strand, AC–H activation approach to the tricyclic core of glionitrin A and B, ACS Omega 7 (14) (2022) 12329–12341. | |
